# AQP4 Aggravates Cognitive Impairment in Sepsis‐Associated Encephalopathy through Inhibiting Na_v_1.6‐Mediated Astrocyte Autophagy

**DOI:** 10.1002/advs.202205862

**Published:** 2023-03-15

**Authors:** Dan‐Dan Zhu, Yue‐Lin Huang, Song‐Yu Guo, Na Li, Xue‐Wei Yang, Ao‐Ran Sui, Qiong Wu, Yue Zhang, Yue Kong, Qi‐Fa Li, Ting Zhang, Wen‐Fei Zheng, Ai‐Ping Li, Jian Yu, Tong‐Hui Ma, Shao Li

**Affiliations:** ^1^ Department of Physiology College of Basic Medical Sciences Liaoning Provincial Key Laboratory of Cerebral Diseases National‐Local Joint Engineering Research Center for Drug‐Research and Development (R & D) of Neurodegenerative Diseases Dalian Medical University Dalian 116044 China; ^2^ Department of Critical Care Medicine the Second Hospital of Dalian Medical University Dalian 116023 China; ^3^ School of Medicine Nanjing University of Chinese Medicine Nanjing 210023 China

**Keywords:** AQP4, astrocyte, autophagy, neuroinflammation, sepsis‐associated encephalopathy, sodium channel Na_v_1.6

## Abstract

The pathology of sepsis‐associated encephalopathy (SAE) is related to astrocyte‐inflammation associated with aquaporin‐4 (AQP4). The aim here is to investigate the effects of AQP4 associated with SAE and reveal its underlying mechanism causing cognitive impairment. The in vivo experimental results reveal that AQP4 in peripheral blood of patients with SAE is up‐regulated, also the cortical and hippocampal tissue of cecal ligation and perforation (CLP) mouse brain has significant rise in AQP4. Furthermore, the data suggest that AQP4 deletion could attenuate learning and memory impairment, attributing to activation of astrocytic autophagy, inactivation of astrocyte and downregulate the expression of proinflammatory cytokines induced by CLP or lipopolysaccharide (LPS). Furthermore, the activation effect of AQP4 knockout on CLP or LPS‐induced PPAR‐γ inhibiting in astrocyte is related to intracellular Ca^2+^ level and sodium channel activity. Learning and memory impairment in SAE mouse model are attenuated by AQP4 knockout through activating autophagy, inhibiting neuroinflammation leading to neuroprotection via down‐regulation of Na_v_1.6 channels in the astrocytes. This results in the reduction of Ca^2+^ accumulation in the cell cytosol furthermore activating the inhibition of PPAR‐γ signal transduction pathway in astrocytes.

## Graphical abstract

SAE mouse model is constructed by cecal ligation and perforation for in vivo experiments and consequent electrophysiology, behavior, and molecular analysis. Primary astrocytes are cultured and stimulated by using lipopolysaccharide (LPS) for in vitro analysis. The pictographic flowchart theorizes that AQP4 aggravates sepsis‐induced neuronal injury and cognitive dysfunction by inhibiting autophagy and activating an inflammatory response in astrocytes.

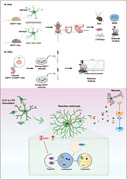

## 1 Introduction

Sepsis associated encephalopathy (SAE) is one of the important public health problems that threaten people's health and affects social development, which needs to be solved urgently.^[^ ^1^, ^2^ ^]^ One of the main clinical manifestations of SAE is cognitive dysfunction,^[^ ^3^, ^4^ ^]^ along with its association with increased morbidity and mortality worldwide.^[^ ^5^ ^]^ Although neuroinflammation,^[^ ^6^, ^7^ ^]^ autophagy,^[^ ^8^, ^9^ ^]^ neuronal injury^[^ ^10^, ^11^ ^]^ are reported as etiological factors of SAE, the potential pathological mechanism related to SAE are very complicated with multiple influencing factors and thus remains to be elucidated.

Astrocyte is considered to be “stewards” of the nervous system performing an important role in the SAE associated neuronal damage.^[^ ^7^, ^12^ ^]^ Synapse modifying factors secreted by astrocytes, such as tumor necrosis factor‐*α* (TNF‐*α*), interleukin‐6 (IL‐6), and interleukin‐1*β* (IL‐1*β*), are necessary for regulation of synaptic plasticity.^[^ ^13^ ^]^ Recently, studies have also found that decreased autophagy level of astrocytes can lead to activation of astrocytes along with increased release of inflammatory factors, which is the main cause of neuronal injury,^[^ ^14^ ^]^ whereas autophagy flux upregulation of astrocytes can improve neuronal activity, reduce neuronal apoptosis and improve neural function.^[^ ^15^ ^]^ Therefore, the mechanism associated with astrocyte autophagy along with its role in relation to SAE is of great clinical significance and should be further researched.

Aquaporin 4 (AQP4) is a selective membrane‐bound water channel having a high expression level in astrocytes. It is not only the gateway that water enters and exits astrocytes, but also a key molecule in astrocytes that can initiate intracellular signaling events to regulate the release of astrocytes inflammatory factors that affect the function of neurons.^[^ ^16^ ^]^ The increase of AQP4 can activate astrocytes, resulting in the secretion of inflammatory factors, which in turn reduces the supporting function of astrocyte towards the neurons, thus making the neurons vulnerable to inflammatory factors.^[^ ^17^ ^]^ In recent years, multiple reports have surfaced regarding the involvement of AQP4 in the pathogenesis of SAE, but its potential mechanism still remains unclear. Some scholars have found that the expression of AQP4 is significantly raised in peripheral blood, cortex, and hippocampus of sepsis patients, along with an enhanced inflammatory response and cognitive dysfunction aggravation.^[^ ^17^, ^18^, ^19^ ^]^ AQP4 knockdown can reduce lipopolysaccharide (LPS) ‐induced astrocyte activation thus decreasing the expression levels of TNF‐*α* and IL‐6.^[^ ^20^ ^]^ However, some scholars also found that AQP4 expression decreased in cortex of SAE mice.^[^ ^12^ ^]^ Therefore, the protein expression level changes and roles of AQP4 in SAE have not been clarified.

The generation of action potential has always been associated with voltage‐gated sodium channels (VGSCs), which are primarily expressed and seen in neurons.^[^ ^21^ ^]^ Out of the expressed VGSCs subtypes in an adult's central nervous system (CNS) Na_v_1.6 is the most abundant, and studies have demonstrated that nonexcitable cells, astrocytes, also express Na_v_1.6.^[^ ^22^, ^23^, ^24^ ^]^ A growing body of evidence points out that these channels not only regulate but also participate in the activation, immunoreactivity and inflammation of astrocytes through signaling mechanisms^[^ ^23^, ^25^, ^26^, ^27^ ^]^ especially modulating intracellular Ca^2+^. In an experimental model of epilepsy, rats show a significant upregulation of Na_v_1.6 in activated astrocyte.^[^ ^28^ ^]^ Exposure to LPS would cause a Na^+^ influx through VGSCs leading to an accumulation of sodium ions in the cytoplasm, which in turn causes the glial cells to be activated and as a consequence an inflammatory pathway is triggered.^[^ ^29^, ^30^ ^]^ An additional study demonstrated that Na_v_1.6 expression was significantly changed in septic patients.^[^ ^31^ ^]^

In this current study, we aim to investigate whether AQP4 deletion has a neuroprotective effect in the cecal ligation and perforation (CLP) mouse model and reveal the mechanism underlying this kind of protection. We found that AQP4 knockout alleviated the cognitive dysfunction and neuronal injury, reduced neuroinflammatory response, glial activation, increased the level of astrocyte autophagy and decreased astrocyte Na_v_1.6 expression in the CLP mice model. In vitro study has revealed that AQP4 knockout reduces LPS‐induced astrocyte activation. This might be due to its anti‐inflammatory effect via down‐regulating astrocyte Na_v_1.6 and subsequent suppression of Ca^2+^ ion build‐up in the cell cytoplasm which furthermore activates the inhibition of PPAR‐*γ* signaling pathway. These aforementioned reports conclusively point out that AQP4 channel could potentially be targeted as a protein of choice for the treatment of SAE.

## 2 Results

### 2.1 AQP4 Expression Was Elevated in Peripheral Blood of Patients and Mice Brain with Sepsis Associated Encephalopathy

Previous studies have shown that AQP4 can be secreted into peripheral blood through brain‐derived plasma exosomes thus elevating AQP4 expression can be detected in peripheral blood, in case of CNS disorders such as Alzheimer's disease and traumatic brain injury and so on. Therefore, AQP4 may be a useful peripheral blood biomarker reflecting changes in brain inflammation.^[^ ^32^, ^33^ ^]^ However, no studies have shown the changes of AQP4 in SAE. To verify the change of AQP4 in SAE, we analyzed peripheral blood samples from 33 SAE patients, 27 sepsis patients and 20 healthy individuals. Gender and age among the three groups had no significant differences (*p* > 0.05) (Table S1, Supporting Information). Furthermore, body temperature, heart rate, SOFA score and 60 d mortality also exhibited no significant difference between the sepsis group and SAE group (*p* > 0.05) (Table S1, Supporting Information). Interestingly, APACHE II score (*p*<0.01), length of the ventilator (*p*<0.05), length of ICU stay (*p*<0.01) and mortality at 28 d (*p*<0.05) in SAE group was significantly higher when compared with the sepsis group. Statistical analysis showed the difference to be significant (Table S1, Supporting Information).

**Figure 1:**
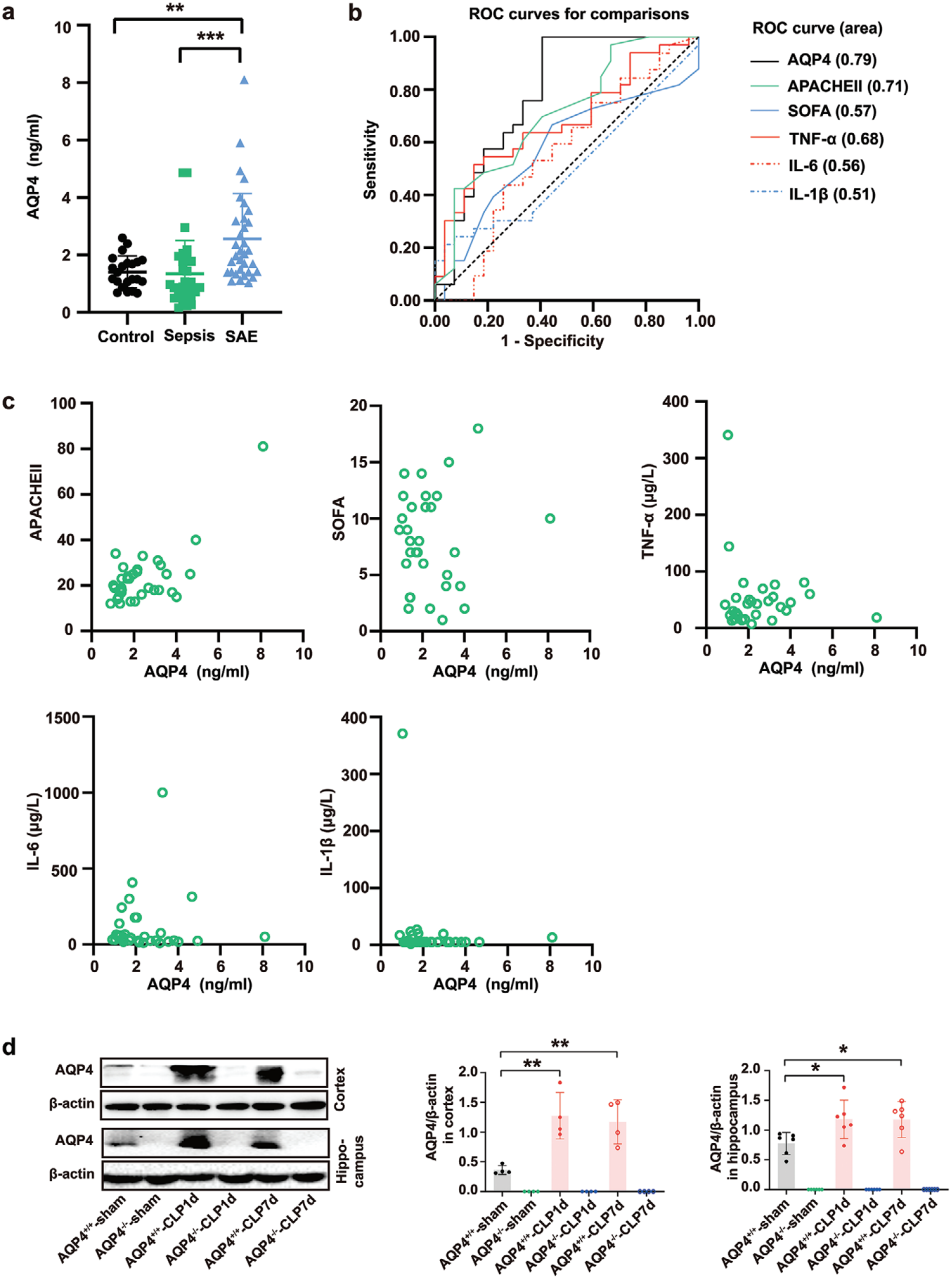
AQP4 expression was elevated in peripheral blood of patients and mice brain with sepsis associated encephalopathy. a) The change of AQP4 levels in peripheral blood of healthy patients, sepsis patients, and SAE patients was detected by ELSA. Healthy controls (*n* = 20) and sepsis patients without encephalopathy (*n* = 27), sepsis related encephalopathy (*n* = 33). Data are presented as the mean ± SD. **p < 0.01, ***p < 0.001, one‐way ANOVA with Tukey's post hoc test. b) The area under ROC curve of SOFA score, APACHE II score, peripheral blood AQP4, and TNF‐*α*, IL‐6, and IL‐1*β* of SAE patients. AQP4 has the largest area under the curve. c) Pearson correlation analysis between AQP4 and SOFA score, APACHE II score, TNF‐*α*, IL‐6, IL‐1*β* in patients with sepsis associated encephalopathy. d) Representative Western blot bands of AQP4 expression levels in cortex and hippocampus of mice. *n* = 4–6 mice for each group. Data are presented as mean ± SD. * *p* < 0.05; ** *p* < 0.01; *** *p* < 0.001; One‐way ANOVA with Tukey's post hoc test.

To clarify the change of AQP4 in peripheral blood of SAE patients, we compared the average AQP4 concentration between the peripheral blood of healthy subjects (1.41 ± 0.56 ng mL^‐1^) and patients with sepsis (1.34 ± 1.16 ng mL^‐1^). However, there was no significant difference between them. Interestingly, AQP4 amount in the SAE patient group (2.56 ± 1.58 ng mL^‐1^; *p*<0.001) were significantly greater compared with the septic patient group (**Figure** **1**a). We further analyzed the area under ROC curve of disease severity score (SOFA score, APACHE II score), peripheral blood AQP4, and inflammatory factors such as TNF‐*α*, IL‐6, and IL‐1*β* of sepsis along with SAE patients (Figure 1b). Compared with other parameters, concentration of AQP4 best differentiated SAE from all other conditions (area under the curve [AUC], 0.79; 95% CI, 0.66–0.91, *p*<0.0001) (Figure 1b). CNS dysfunction in SAE may manifest as abnormalities in levels of biomarkers of CNS injury (such as neuron‐specific enolase [NSE] and S100*β* neurofilament light chain).^[^ ^34^ ^]^ Our study also investigated the levels of NSE and S100*β* in the serum of patients with sepsis or SAE. Both NSE and S100*β* levels in SAE patients were significantly higher than in septic patients without SAE (21.73 ± 11.11 ng mL^‐1^ versus 14.89 ± 5.26 ng mL^‐1^, *p* < 0.01; 0.38 ± 0.23 ng mL^‐1^ versus 0.12 ± 0.09 ng mL^‐1^, *p* < 0.0001) (Figure S1a,b, Supporting Information). Of note, there was statistically significant positive correlation between AQP4 and APACHE II score (*r* = 0.72, *p* < 0.0001), AQP4 and NSE score (*r* = 0.45, *p* < 0.01), AQP4 and S100*β* score (*r* = 0.55, *p* < 0.001), but there was no statistically significant correlation between AQP4 and SOFA score, TNF‐*α*, IL‐6 and IL‐1*β* (Figure 1c, Figure S1c,d, Supporting Information).

Considering the changes of AQP4 in the peripheral blood of SAE patients, we speculated that AQP4 plays an important role in brain injury of SAE, so we made a CLP mouse model to observe the changes of AQP4 in the brain. We found that the AQP4 protein levels in cortex and hippocampus were remarkably higher in the CLP group as compared to the sham surgical group at day 1 and day 7 after surgery (Figure 1d).

### 2.2 AQP4 Deletion Improved Survival Rate and Ameliorated Brain Injury in CLP‐Induced Sepsis Mice

**Figure 2:**
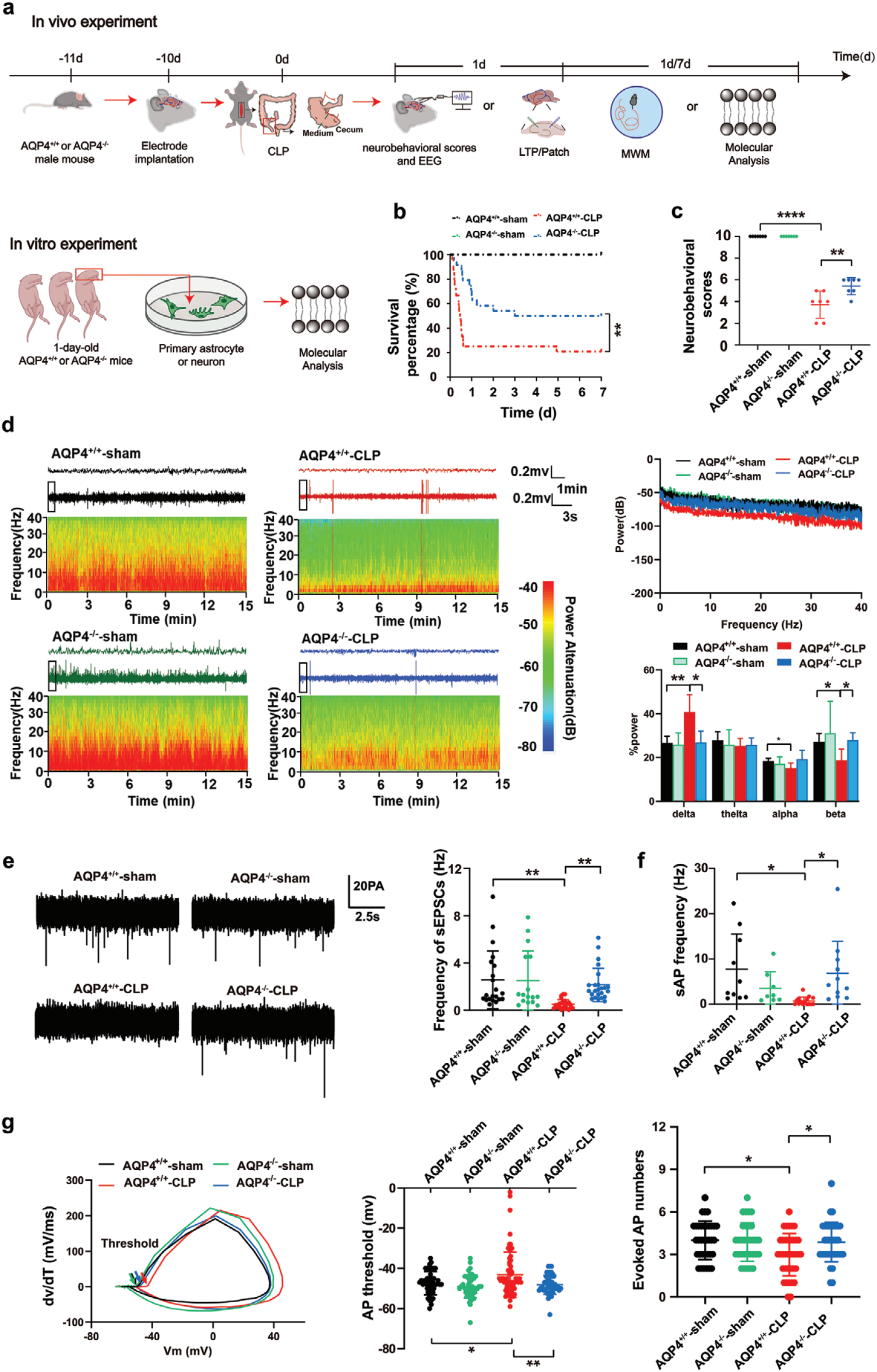
AQP4 deletion improved survival rate and ameliorated sepsis‐induced neurologic injury in brain of CLP‐induced sepsis in mice. a) The program of septic model preparation and arrangement of EEG and neurological score in the present study. b) The survival curve analysis is of the survival rates representing each group mice after modeling. *n* = 24 mice for each group. c) The neurobehavioral score which reflects the neurological injury of mice. *n* = 7 mice for each group. d) Relative EEG analysis of different groups of mice included EEG spectrum (left), EEG average power spectrum (upper right), average power percentage of *α*, *β*, *δ*, *θ* waves (lower right), *n* = 4–6 mice per group. e) Spontaneous EPSCs were recorded. The representative sEPSC traces and quantification of sEPSC frequency are shown in (e). Neurons from 6 mice per group. f) Spontaneous action potential was recorded and quantification of sAP frequency is shown in (f). Neurons from 6 mice. g) The 1st derivative of the somatic membrane voltage (d*V*/d*t*) versus membrane voltage (*V*_m_) in phase plot. The arrow points to the action potential voltage threshold (left). Quantification of evoked AP thresholds (middle) and quantification of evoked AP numbers (right). Neurons from 6 mice. b) Log‐rank (Mantel‐Cox) test was used. c–g) Data are presented as mean ± SD. * *p* < 0.05, **p < 0.01, ****p < 0.0001; two‐way ANOVA with Tukey's post hoc test.

Furthermore, in order to show the participation of AQP4 in SAE's pathological process we used AQP4 knockout mice in our experiments.^[^ ^31^ ^]^ The AQP4^−/−^ mice were confirmed by PCR genotyping (Figure S2a, Supporting Information), and did not express AQP4 protein and mRNA separately (Figure S2b–d, Supporting Information). **Figure** **2**a shows the experimental procedure for septic mice model. All of the sham surgery animals had a 100% survival rate with normal behavior throughout the 7 d study period. However, mice in the CLP group, post‐sepsis showed survivability of 20.8% (5 of 24 mice survived) by day 7. AQP4^−/−^‐CLP group mice exhibited a lower mortality revealing a better survival rate (12 of 24 mice survived) compared to AQP4^+/+^‐CLP group mice (Figure 2b). The neurobehavioral results of the AQP4^−/−^‐CLP mice had significantly higher scores than those of the AQP4^+/+^‐CLP mice (5.14 ± 0.90 in AQP4^−/−^‐CLP mice, 3.71 ± 1.25 in AQP4^+/+^‐CLP mice, *p* < 0.01) (Figure 2c). To examine neuro‐excitability, mice were subjected to electroencephalogram (EEG) monitoring at day 1 after surgery. The energy spectra demonstrated that CLP reduced cortex EEG activity, whereas AQP4^−/−^alleviated the inhibition of septic mice brain electrical activity (Figure 2d). The analysis of power spectrum revealed that septic mice show a low power spectrum than sham mice (AQP4^+/+^‐CLP: ‐85.15 ± 8.28; AQP4^+/+^‐sham: 66.23 ± 7.48; *p* < 0.0001; Figure 2d), and AQP4 knock out mice displayed an increase of power spectrum (AQP4^−/−^‐CLP: 72.78 ± 8.69; AQP4^+/+^‐CLP: ‐85.15 ± 8.28; *p* < 0.0001; Figure 2d), compared with septic mice. And the EEG power bands (delta, alpha, and beta) also showed significantly diverse distribution (Figure 2d). Alpha and beta waves’ activities were decreased and delta wave activity was increased during the observation period in AQP4^+/+^‐CLP mice, on which these results were found to be significantly different, however AQP4 knock out can reverse those changes (Figure 2d).

To investigate the neuronal excitability changes in the mice brain during CLP, we studied the effect of CLP on the frequency of sEPSCs using patch clamp in mouse brain slices. The mean frequency of sEPSCs was significantly decreased in AQP4^+/+^‐CLP mice relative to AQP4^+/+^‐sham mice (Figure 2e, right; *p* < 0.01). Compared with the AQP4^+/+^ mice, the AQP4^−/−^ mice showed an increased mean frequency of sEPSCs in the CLP model (*p* < 0.01), but there were no significant compared with AQP4^−/−^‐sham mice (Figure 2e, right). The average peak amplitude of sEPSCs did not differ in mice from the four groups (Figure S3a, Supporting Information; *p* > 0.05). The mean frequency of sAP and numbers of eAP were significantly decreased, eAP threshold was elevated in AQP4^+/+^‐CLP mice relative to AQP4^+/+^‐sham mice (Figure 2f,g). Compared to AQP4^+/+^ mice, the AQP4^−/−^ mice showed an increased mean frequency of sAP and numbers of eAP, decreased eAP threshold in the CLP model (Figure 2f,g). While sAP mean peak amplitude, eAP membrane potential, eAP mean peak amplitude and half wave width of eAP did not differ between groups in sepsis (Figure S3b,c, Supporting Information; *p* > 0.05). These data indicated that AQP4 deletion improved survival rate and ameliorated sepsis‐induced brain neurologic injury in mice with CLP‐induced sepsis.

### 2.3 AQP4 Knock Out Ameliorated Cognitive Dysfunction and Improves Synaptic Plasticity of CLP‐Induced Sepsis in Mice

**Figure 3:**
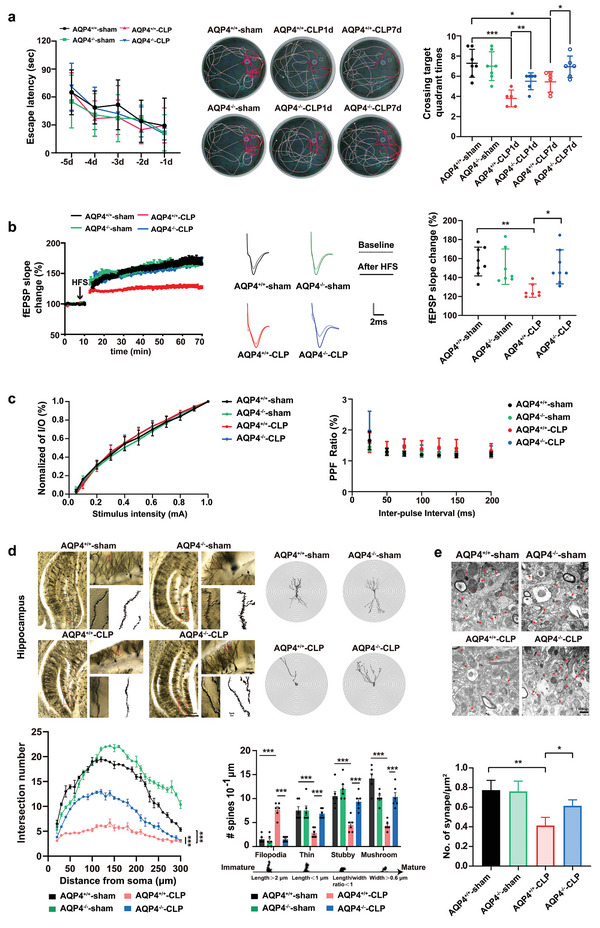
AQP4 knock out ameliorated cognitive dysfunction and improves synaptic plasticity of CLP‐induced sepsis in mice. a) Mice were subjected to the Morris water maze test. Left, the mean escape latency; middle, tracings of the typical swim patterns; right, crossing target quadrant times by the mice. *n* = 5–7 mice for each group. b) Left, the effects of HFS on the fEPSP initial slope (HFS, high frequency stimulation. *n* = 7–8 mice per group). Middle, representative fEPSP traces for data shown. Right, Cumulative data showing the mean fEPSP slope 60 min post‐HFS. *n* = 7–8 mice per group. c) Left, cumulative data showing the normalized I/O. Right, cumulative data showing the PPF ratio. *n* = 5 mice per group, 4–5 slices per animal. d) Upper panel, representative dendritic spines in hippocampus of four groups (scale bar, 500 µm, 50 µm, 1 µm); lower left panel, AQP4 knockout in septic mice increases apical node and spines in hippocampus, while AQP4^+/+^‐CLP shows no such change (at least 10 neurons from six mice per group were analyzed by the Sholl); lower right panel, statistical analysis showed the effect of AQP4 knockout in septic mice on dendritic spines. e) Upper panel, representative transmission electron micrographs of synapses in cortex (red arrows: synapses; scale bar, 500 nm); lower panel, statistical analysis of the densities of synapses. Data are presented as mean ± SD. Data in a (Left) was analyzed by repeated‐measures ANOVA with Tukey's post hoc test. a) (Right) One‐way ANOVA with Tukey's post hoc test. b–e) Two‐way ANOVA with Tukey's post hoc test. * *p* < 0.05, ** *p* < 0.01, *** *p* < 0.001, *** *p* < 0.0001.

The main features of SAE are cognitive deficits. To find out whether AQP4 has any impact on learning and memory, Morris water maze test was carried at day 1 and day 7 post‐CLP surgery, prior to animal sacrifice. There was no significant difference in the acquisition phase of learning (a latency to find the platform) among four groups (**Figure** **3**a left; *F*(12, 115) = 0.38, *p* > 0.05). Representation of swimming trace routes of mice is shown in Figure 3a (middle). Crossing target quadrant times by AQP4^+/+^‐sham surgery mice were significantly greater than that of AQP4^+/+^‐CLP mice at day 1 and day 7 post sepsis onset. In contrast, AQP4 knock out mice remarkably overcame sepsis induced spatial working memory deficits as compared to the AQP4 wild‐type CLP group mice (Figure 3a right). These results demonstrated that the AQP4 knock out prominently ameliorated CLP‐induced hippocampus‐dependent cognitive dysfunction (learning and memory function) in the septic mice.

Long‐term potentiation (LTP) is widely accepted as a major cellular mechanism and memory.^[^ ^35^ ^]^ Thus we further recorded hippocampal LTP on the day 1 post sepsis onset providing the underlying mechanism of learning and memory.^[^ ^36^ ^]^ Data from the results showed a suppression of LTP in the septic mice, indicated by a significant lowering in the fEPSP slope (Figure 3b), which might be the cause of memory retrieval dysfunction. AQP4 knock out has better apparent effects on the alleviation of LTP inhibition as seen in septic mice (Figure 3b). At the same time, the normalized I/O and PPF in the hippocampus were immediately measured with no significant difference when compared among the four groups (Figure 3c).

Sepsis induced mice exhibit a reduction in dendritic spines of the hippocampus contributing to LTP and cognition impairment.^[^ ^10^ ^]^ The structural synaptic plasticity was verified by measuring the postsynaptic dendrite complexity alterations. Dendritic spine density and the morphology of hippocampus were seen by using Golgi staining (Figure 3d). Utilizing concentric circle (Sholl's) analysis, showed a significant increase in the neurite arborization, spine generation and maturation in the AQP4^−/−^‐CLP mice as compared to AQP4^+/+^‐CLP mice, suggesting an augmented postsynaptic plasticity in AQP4^−/−^‐CLP mice (Figure 3d). These results reveal AQP4 knockout have an improved effect on the impairments of synaptic plasticity seen in septic mice. In the septic mice, the synapse was decreased significantly (Figure 3e). In the AQP4^−/−^‐CLP mice, the synapse density was preserved (Figure 3e). Postsynaptic protein NMDA receptors (NR2B) and postsynaptic density 95 (PSD95) are proteins related to synaptic plasticity.^[^ ^37^, ^38^ ^]^ Thus, we detected the expression level of NR2B and PSD95 in cortex and hippocampus to evaluate cognitive impairment of mice. In consistence with the above results, levels of NR2B and PSD95 were decreased significantly in the cortex and hippocampus of AQP4^+/+^‐CLP mice at day 1 and day 7, while the levels of these proteins recovered in AQP4^−/−^‐CLP mice (Figure S4, Supporting Information). These results demonstrated that AQP4 knock out ameliorated cognitive dysfunction and improves synaptic plasticity of CLP‐induced sepsis in mice.

### 2.4 AQP4 Deletion Diminished Astrocyte Activation and Mitigated the Inflammatory Cytokine Response in Septic Mice

**Figure 4:**
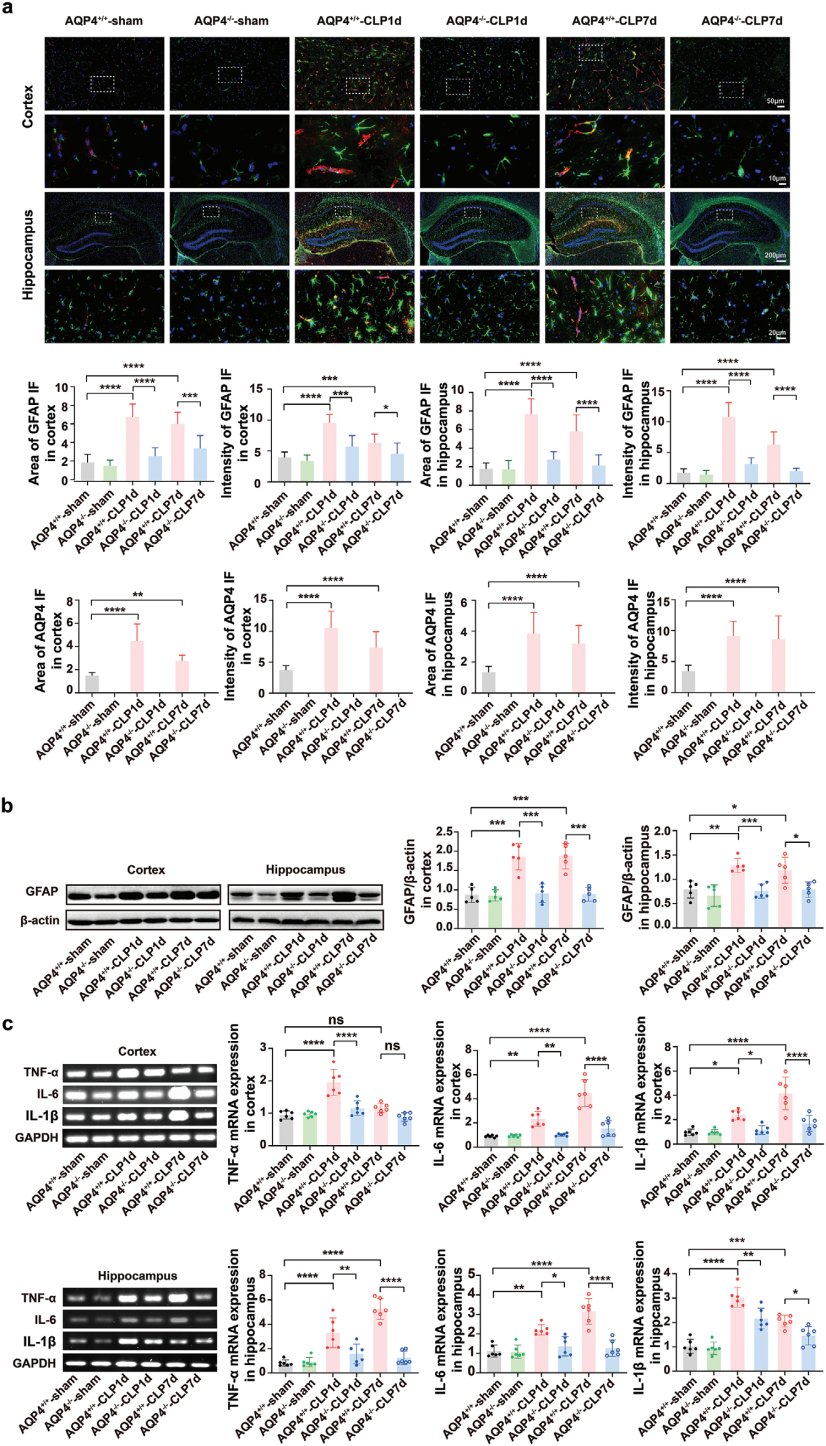
AQP4 knockout diminished astrocyte activation and mitigated the inflammatory cytokine response in septic mice. a) Immunofluorescence of GFAP^+^(green) astrocytes and AQP4 (red) in cortex and hippocampus of mice brain slice(upper), different magnification scale bar respectively: 50 µm; 10 µm; 200 µm; 20 µm. Middle and lower panel, quantification of area and intensity of GFAP and AQP4 in the mice cortex and hippocampus among different groups. *n* = 9 mice for each group. b) Representative Western blot bands of the GFAP expression levels in cortex and hippocampus of mice (left); right panel, quantification of GFAP/*β*‐actin in the mice cortex and hippocampus among different groups. *n* = 5 mice for each group. c) Representative RT‐PCR bands of the TNF‐*α*, IL‐6, IL‐1*β* mRNA expression levels in cortex of mice (upper left); upper right, quantification of TNF‐*α*, IL‐6, IL‐1*β* in the mice cortex was done and normalized to the mRNA level of GAPDH among different groups. Representative RT‐PCR bands of the TNF‐*α*, IL‐6, IL‐1*β* mRNA expression levels in hippocampus of mice (lower left); lower right, quantification of TNF‐*α*, IL‐6, IL‐1*β* was done and normalized to the mRNA level of GAPDH in the mice hippocampus among different groups. *n* = 6 mice for each group. Data are presented as mean ± SD. * *p* < 0.05, ** *p* < 0.01, *** *p* < 0.001, **** *p* < 0.0001; one‐way ANOVA with Tukey's post hoc test.

Astrocyte activation is one of the main neuropathological features of SAE. Astrocytes secrete pro‐inflammatory factors that can induce and/or regulate inflammatory response magnitude and outcome, thus controlling neuroinflammation in sepsis.^[^ ^39^, ^40^ ^]^ We investigated the effects of AQP4 on the CLP‐induced astrocyte responses especially to observe its activation, therefore western blot and immunofluorescence staining were done. RT‐PCR was performed to evaluate the level of inflammatory cytokines. Evaluation of astrocyte immunoreactivity was done by GFAP and AQP4 in the cortex and hippocampus. In cortical and hippocampal tissue of septic mice, an enlarged cell body with thick, shrunk processes was shown in GFAP‐positive cells, which were consistent with the morphology of an activated astrocyte. Conversely, GFAP‐positive cells in the sham surgery group exhibit a thin cell body with fine and long processes which again is consistent with the morphological ramifications of a resting astrocyte. GFAP expression measurement showed that the area along with the intensity of GFAP positive cells were significantly increased in AQP4^+/+^‐CLP mice compared to sham surgery mice in the cortex and hippocampus (**Figure** **4**a). In AQP4^−/−^‐CLP mice, the area and intensity of GFAP‐positive cells in the cortex and hippocampus were dramatically decreased (Figure 4a). We also found that the area and intensity of AQP4‐positive cells in cortex and hippocampus were remarkably decreased in the AQP4^−/−^‐CLP mice as compared to the AQP4^+/+^‐CLP mice on the day 1 and day 7 after surgery (Figure 4a). Similarly, the GFAP protein expression was increased in septic mice, while decreased in AQP4^−/−^‐CLP mice (Figure 4b).

Cytokines such as TNF‐*α*, IL‐6, IL1‐*β* represent astrocyte‐derived factors along with proinflammatory factors’ activity.^[^ ^41^ ^]^ Thus, inflammatory cytokines levels in cortex and hippocampus were measured in terms of mRNA. Both cortical and hippocampal tissue showed elevated levels of IL‐6 and IL‐1*β* at day 1 and day 7 post‐surgery, however the levels of these inflammatory cytokines dramatically decreased in AQP4 knockout mice tissue at the aforementioned both time points (Figure 4c). The levels of TNF‐*α* in both cortex and hippocampus were elevated at day 1 post‐surgery and were dramatically decreased in AQP4 knockout mice, but not at day 7 after sepsis onset in cortical tissue (Figure 4c). These results indicated that AQP4 deletion diminished astrocyte activation and mitigated the inflammatory cytokine response in septic mice.

### 2.5 AQP4 Deletion Promotes Astrocytic Autophagy via Activation of PPAR‐*γ*/mTOR Signaling Pathway in Septic Mice Brain

**Figure 5:**
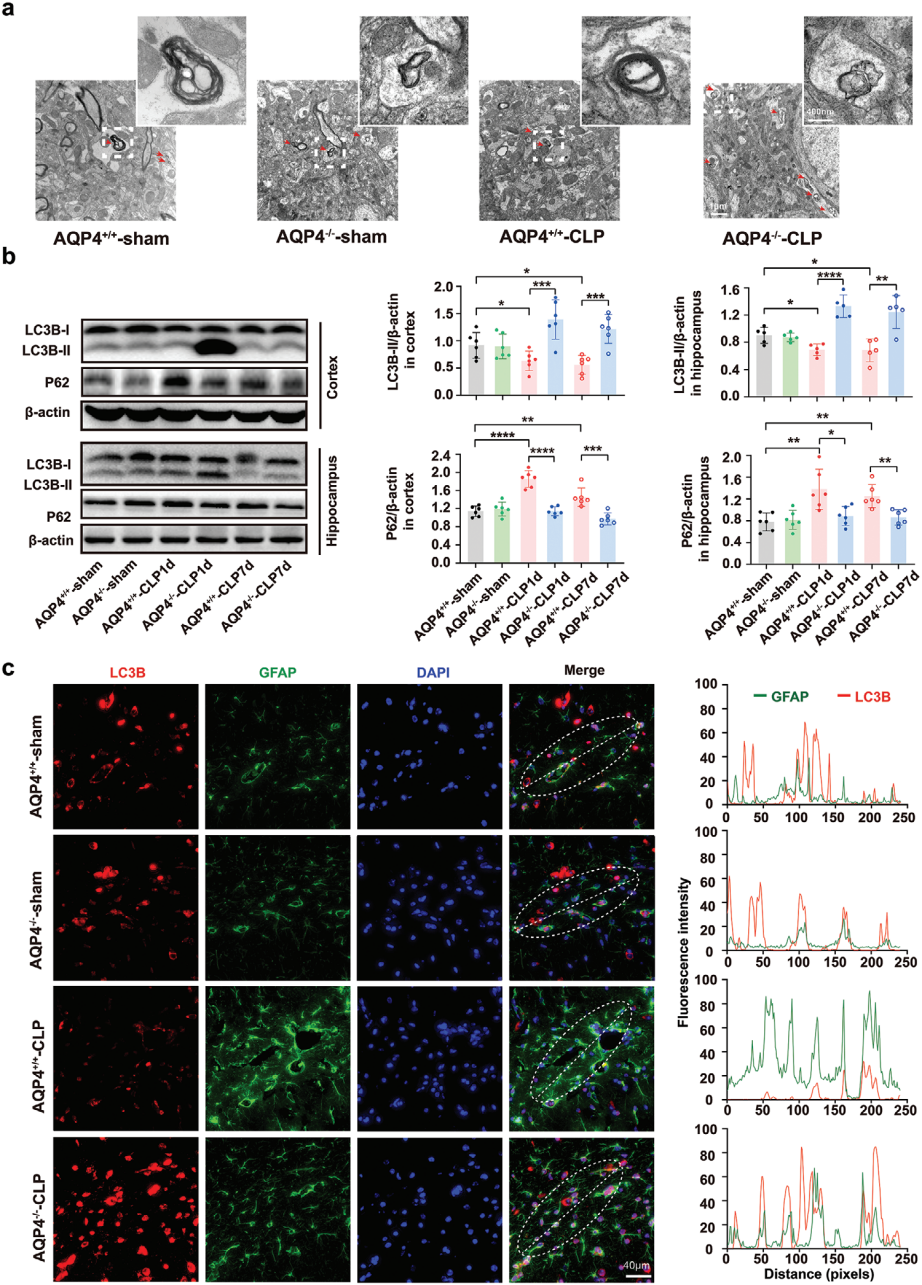
AQP4 knockout restored autophagy in septic mice brain. a) Representative transmission electron micrographs of the hippocampus (Scale bar, 200 µm) of full image (Scale bar, 1 µm). Red arrows indicate autophagosome. b) Representative Western blot bands of the LC3B‐II, LC3B‐I, and p62 expression levels in cortex and hippocampus of mice(left); right panel, quantification of LC3B‐II/*β*‐actin and p62/*β*‐actin in the mice cortex and hippocampus among different groups. *n* = 5–6 mice for each group. Data are presented as mean ± SD. * *p* < 0.05, ** *p* < 0.01, *** *p* < 0.001, **** *p* < 0.0001; one‐way ANOVA with Tukey's post hoc test. c) Brain slice of each group mice were immunostained with LC3B (red) and GFAP (green) (complete co‐localization) in the hippocampal CA1 region (left), scale bar, 40 µm; right panel, quantitative analysis of intensity of LC3B puncta and GFAP immunofluorescence.

According to certain literatures the pathogenesis of SAE often involves autophagy. In our present study, transmission electron microscopy analysis revealed lesser number of autophagosomes in AQP4^+/+^‐CLP mice hippocampus as compared to the AQP4^+/+^‐sham mice. The AQP4^−/−^ mice that underwent CLP shows an even higher number (**Figure** **5**a). Similarly, the cortex and hippocampus of AQP4^+/+^‐CLP mice exhibit a lower LC3B‐II levels than the AQP4^+/+^‐sham mice; however, these parameters were increased in AQP4^−/−^‐CLP mice (Figure 5b). AQP4^+/+^‐CLP mice showed significantly higher amount of p62 in the cortex and hippocampus than in the control group. These CLP‐induced changes were alleviated in AQP4^−/−^ mice (Figure 5b). AQP4 mainly expressed at astrocyte in CNS, thus to further assess whether the astrocyte activation was caused by the inhibition of astrocyte autophagy, we performed double immunofluorescence staining of LC3B and GFAP in brain sections of mice, respectively. AQP4 knockout mice exhibited an increased level of colocalization among LC3B with GFAP, meanwhile the intensity of GFAP was decreased in mice underwent CLP (Figure 5c).

**Figure 6:**
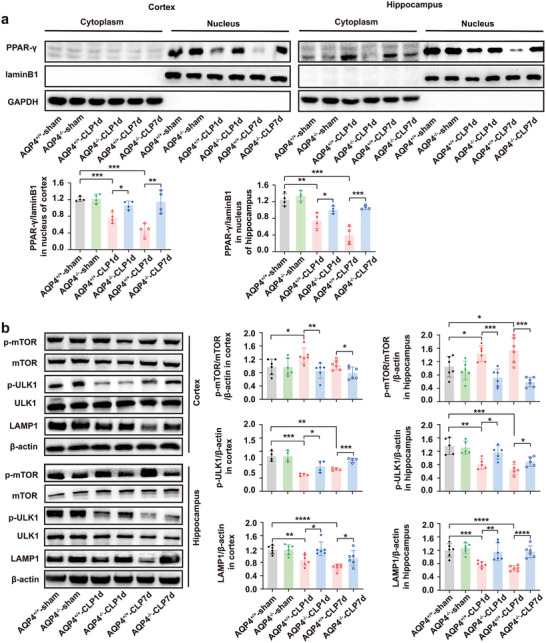
AQP4 knockout activated the PPAR‐*γ*/mTOR signaling pathway in septic mice. a) Representative of Western blot probing for the PPAR‐*γ* entry into the nucleus in the cortex and hippocampus of each group mice (upper panel) and quantitative analysis of PPAR‐*γ* expression levels in the nucleus (lower paned). n = 4 mice for each group. b. Representative Western blot band for the p‐mTOR, mTOR, p‐ULK1, ULK1, LAMP1, *β*‐actin (left) and quantitative analysis of those protein levels (right). *n* = 4–6 mice for each group. Data are presented as mean ± SD. *p < 0.05, ** *p* < 0.01, *** *p* < 0.001, **** *p* < 0.0001; one‐way ANOVA with Tukey's post hoc test.

The ERK1/2, JNK, P38, AKT and GSK3*β* signaling pathways have been found to be involved in autophagy modulation.^[^ ^18^, ^19^ ^]^ We found that CLP did not change the phosphorylated ERK1/2, JNK, P38, AKT and GSK3*β* levels in mice brain (Figure S5, Supporting Information). Our data point out that the inhibition of autophagy by AQP4 had no relationship to the ERK1/2, JNK, P38, AKT and GSK3*β* signaling pathways. Recently study show that AQP4 deletion upregulates PPAR‐*γ* expression and attenuates proinflammatory cytokine release. Peroxisome proliferators‐activated receptor‐*γ* (PPAR‐*γ*), a transcription factor, can regulate mTOR kinase activity, then further activates proteins such as Unc‐51‐like kinase 1(ULK1) which are related to autophagy and lysosomal associated membrane protein 1 (LAMP1) to regulate autophagy.^[^ ^42^, ^43^, ^44^ ^]^ However, whether the PPAR‐*γ*/mTOR pathway can regulate astrocyte autophagy in SAE has not been reported. Recent studies have shown that AQP4 knockout can activate PPAR‐*γ* expression and reduce the release of inflammatory cytokines in the brain.^[^ ^45^ ^]^ Therefore, we detected the expression of PPAR‐*γ* in the nucleus by cytoplasm‐nuclear protein extraction kit and found that AQP4 knockout significantly increased the nuclear expression of PPAR‐*γ* in septic mice cortex and hippocampus (**Figure** **6**a). The expression of p‐mTOR/mTOR ratio was lower in AQP4^−/−^‐CLP mice than in AQP4^+/+^‐CLP mice, and the expression of p‐ULK1 and LAMP1 were higher in AQP4^−/−^‐CLP mice than in AQP4^+/+^‐CLP mice (Figure 6b). These results suggest that AQP4 knockout promotes astrocytic autophagy via activation of PPAR‐*γ*/mTOR signaling pathway in septic mice brain.

### 2.6 AQP4 Deletion Activates PPAR‐*γ*/mTOR‑Dependent Autophagy and Inhibits Inflammation Response in Primary Cultured Astrocytes Treated with LPS

**Figure 7:**
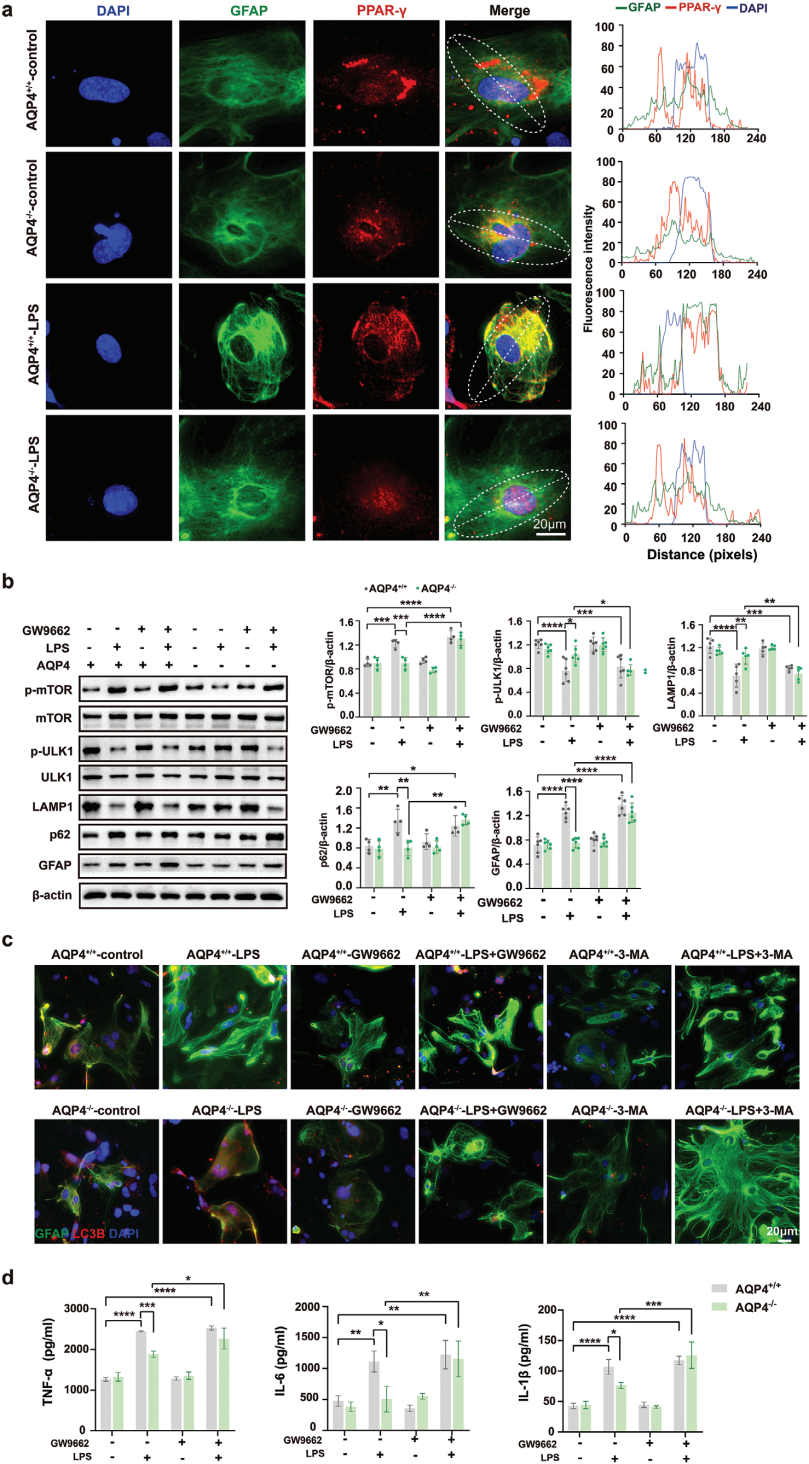
AQP4 deletion activates PPAR‐*γ*/mTOR‑dependent autophagy and inhibits inflammation response in primary cultured astrocytes treated with LPS. a) Primary astrocytes were immunostained with GFAP (green), DAPI (blue), PPAR‐*γ* (red) simultaneously, scale bar, 20 µm (left); right panel, quantitative analysis of GFAP and DAPI, PPAR‐*γ* immunofluorescence intensity. b) The protein levels of p‐mTOR, p‐ULK1, LAMP1, p62, GFAP were determined by Western blot in AQP4^+/+^ and AQP4^−/−^ astrocytes treated with LPS (left) and quantitative analysis of those protein levels, *n* = 4–6 for each group (right). c) Primary astrocytes were immunostained with GFAP (green), DAPI (blue), LC3B (red) simultaneously and the AQP4^+/+^ and AQP4^−/−^ astrocytes treated with LPS, GW9662 or 3‐MA, scale bar, 20 µm. d) The inflammatory cytokines expression levels of TNF‐*α*, IL‐6, IL‐1*β* were determined in astrocyte culture media by ELSA in AQP4^+/+^ and AQP4^−/−^ astrocytes treated with LPS or GW9662. *n* = 3 for each group. b,d) Data are presented as mean ± SD. * *p* < 0.05, ** *p* < 0.01, *** *p* < 0.001, **** *p* < 0.0001; one‐way ANOVA with Tukey's post hoc test.

In order to further evaluate the expression of PPAR‐*γ* in the astrocyte nucleus, double immunofluorescence staining of PPAR‐*γ* and GFAP was performed in primary astrocytes respectively. AQP4 deletion resulted in an increased level of colocalization of PPAR‐*γ* with DAPI, meanwhile the intensity of GFAP was decreased in astrocyte underwent LPS (**Figure** **7**a). Administration of LPS triggered a significant increase in levels of p‐mTOR, p62, and GFAP; a decrease in levels of p‐ULK1 and LAMP1 in primary cultured astrocytes (Figure 7b). Such effects were subdued by AQP4 knockout. Conversely, the PPAR‐*γ* antagonist mitigated the effects of AQP4 knockout on autophagy (Figure 7b). Immunofluorescence image showed AQP4^−/−^‐LPS group LC3B expression increased and GFAP expression decreased compared with AQP4^+/+^‐LPS, however pretreatment with autophagy inhibitor 3‐MA or PPAR‐*γ* antagonist GW9662 could mitigate the effect knockout AQP4 on LPS‐induced primary astrocyte autophagy and activation (Figure 7c). ELISA results showed that the release of TNF‐*α*, IL‐6 and IL‐1*β*, which were previously elicited by LPS were significantly attenuated by AQP4 knockout. Conversely, GW9662 mitigated the effects of AQP4 knockout on the production of TNF‐*α*, IL‐6 and IL‐1*β* (Figure 7d). Collectively, these results reveal that in vitro, AQP4 suppressed astrocyte autophagy and activated astrocyte, which is likely through inhibition of the PPAR‐*γ*/mTOR signaling pathway.

### 2.7 Na_v_1.6 Activation Is Necessary for AQP4‐Mediated Regulation of PPAR‐*γ* Transportation to the Nucleus in Primary Cultured Astrocytes

**Figure 8:**
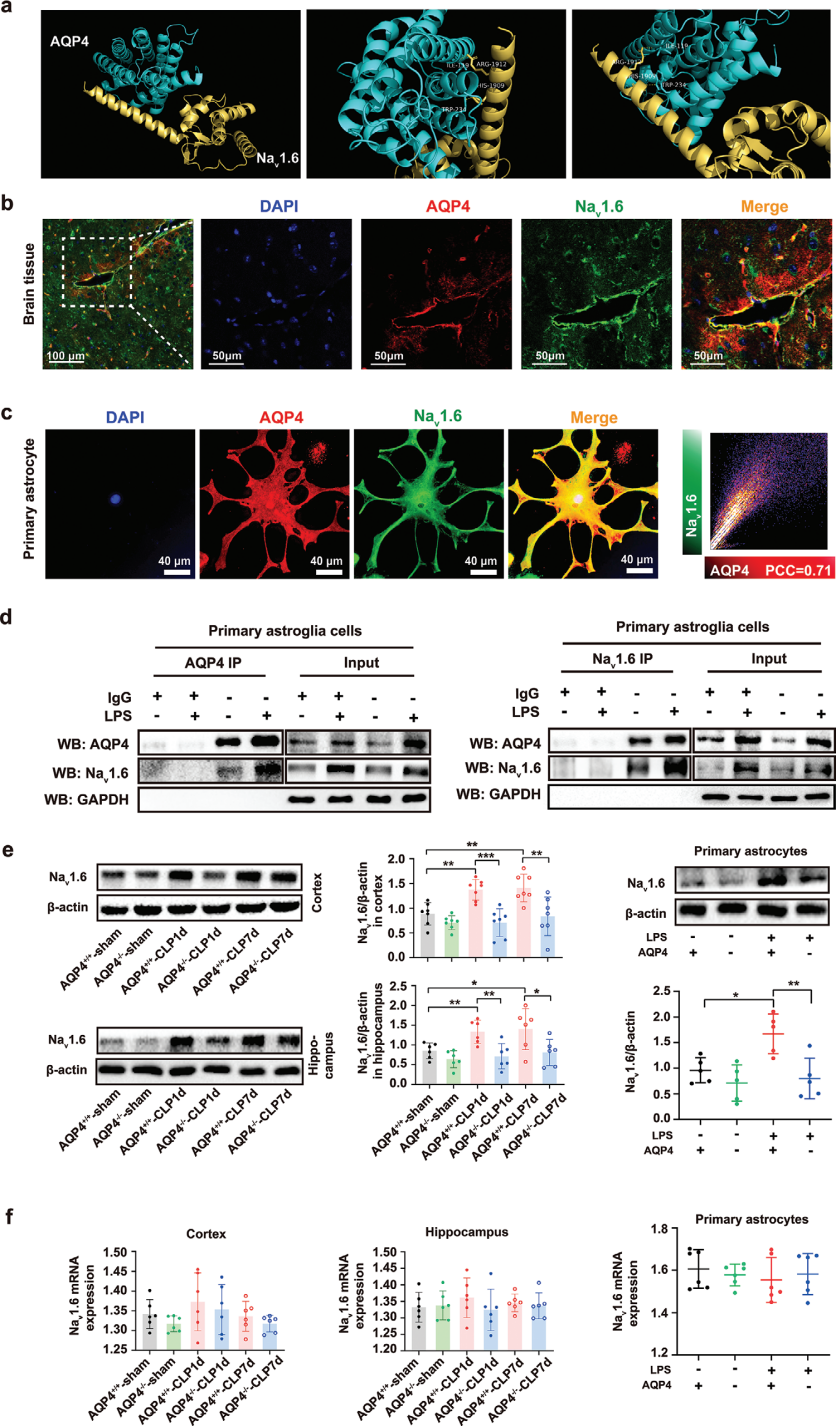
The interaction between AQP4 and Na_v_1.6 in astrocytes. a) AQP4 was docked into the binding site of the Na_v_1.6. surface mode. The AQP4 was represented with green lines; Nav1.6 was represented with yellow lines; the hydrogen bond was shown in dotted yellow line. b) Immunofluorescence for colocalization of AQP4 (red) and Na_v_1.6 (green) in brain slice, scale bar, 100 µm, 50 µm. c) Left, immunofluorescence for colocalization of AQP4 (red) and Na_v_1.6 (green) in primary astrocytes, scale bar, 40 µm. Right, scatterplot of AQP4 (red) and Na_v_1.6 (green) pixel intensities of the astrocyte. d) AQP4 coimmunoprecipitates (IP) with Na_v_1.6. The AQP4^+/+^ and AQP4^−/−^ primary astrocytes treated with LPS. Total proteins were extracted and immunoprecipitated with anti‐AQP4 antibody beads (left). And total proteins were extracted and immunoprecipitated with anti‐Na_v_1.6 antibody beads (right). Immunoprecipitates and total protein extracts (input) were immunoblotted with appropriate antibodies as described in the figure. The input represents the total protein extract used in IP. IP, immunoprecipitation; IgG, negative control. e) Left, representative Western blot band for the Na_v_1.6 in cortex and hippocampus of each group of mice and quantitative analysis of those protein levels, *n* = 6–7 mice for each group. Right, the Western blot band for the Na_v_1.6 (upper right) in AQP4^+/+^ and AQP4^−/−^ primary astrocytes treated with LPS or not and quantitative analysis of this protein levels, *n* = 5 (lower right). f) The mRNA levels of Na_v_1.6 was determined by qPCR in cortex and hippocampus of each group of mice, *n* = 6 mice for each group (left, middle) and in AQP4^+/+^ and AQP4^−/−^ primary astrocytes treated with LPS or not, *n* = 6 for each group(right). e,f) Data are presented as mean ± SD. * *p* < 0.05, ** *p* < 0.01, *** *p* < 0.001; one‐way ANOVA with Tukey's post hoc test.

Na_v_1.6 is one of the predominant subtypes expressed in astrocyte among the voltage‐gated sodium channels (VGSCs) that is involved in regulating immune response.^[^ ^22^, ^23^, ^28^ ^]^ Previously, we have found that Na_v_1.6 plays an important role in regulating inflammation.^[^ ^30^ ^]^ Interestingly, the preferable binding site of AQP4 and Na_v_1.6 was found by molecular docking‐based calculation. The theoretical binding mode of AQP4 and Na_v_1.6 in the binding site of their carbon chain was illustrated in **Figure** **8**a. Importantly, two key hydrogen bond interactions were observed between the ILE‐119, TRP‐234 of AQP4 and the carbonyl group of ARG1912, HIS‐1909 in Na_v_1.6 (Figure 8a). Brain slice immunofluorescence staining and primary astrocytes also showed that AQP4 strongly colocalized with Na_v_1.6 (Figure 8b,c). It could be clearly seen that the AQP4 had a well overlap with Na_v_1.6 (overlap *R* = 0.96), and the *R* (Pearson's correlation coefficients, PCC) value reached 0.71, which proves that AQP4 could well target the Na_v_1.6 in primary astrocyte (Figure 8c). We further demonstrated the impact of LPS on Na_v_1.6 activation using a co‐immunoprecipitation (Co‐IP) assay. The results showed that LPS increased the binding capacity of AQP4 and Na_v_1.6 in primary astrocytes lysates (Figure 8d). The western blot results demonstrated that the expression of Na_v_1.6 in cortex and hippocampus were increased, whereas AQP4 knockout significantly attenuated CLP‐induced up‐regulation of Na_v_1.6 (Figure 8e). Post LPS treatment the AQP4 deletion on Na_v_1.6 expression was further confirmed by observing the primary astrocytes. Western blot analysis confirmed that administration of LPS increased the Na_v_1.6 protein level in astrocyte, which was further attenuated by AQP4 knockout (Figure 8e). However, there was no significant difference in mRNA level among groups in vitro and in vivo (Figure 8f)

**Figure 9:**
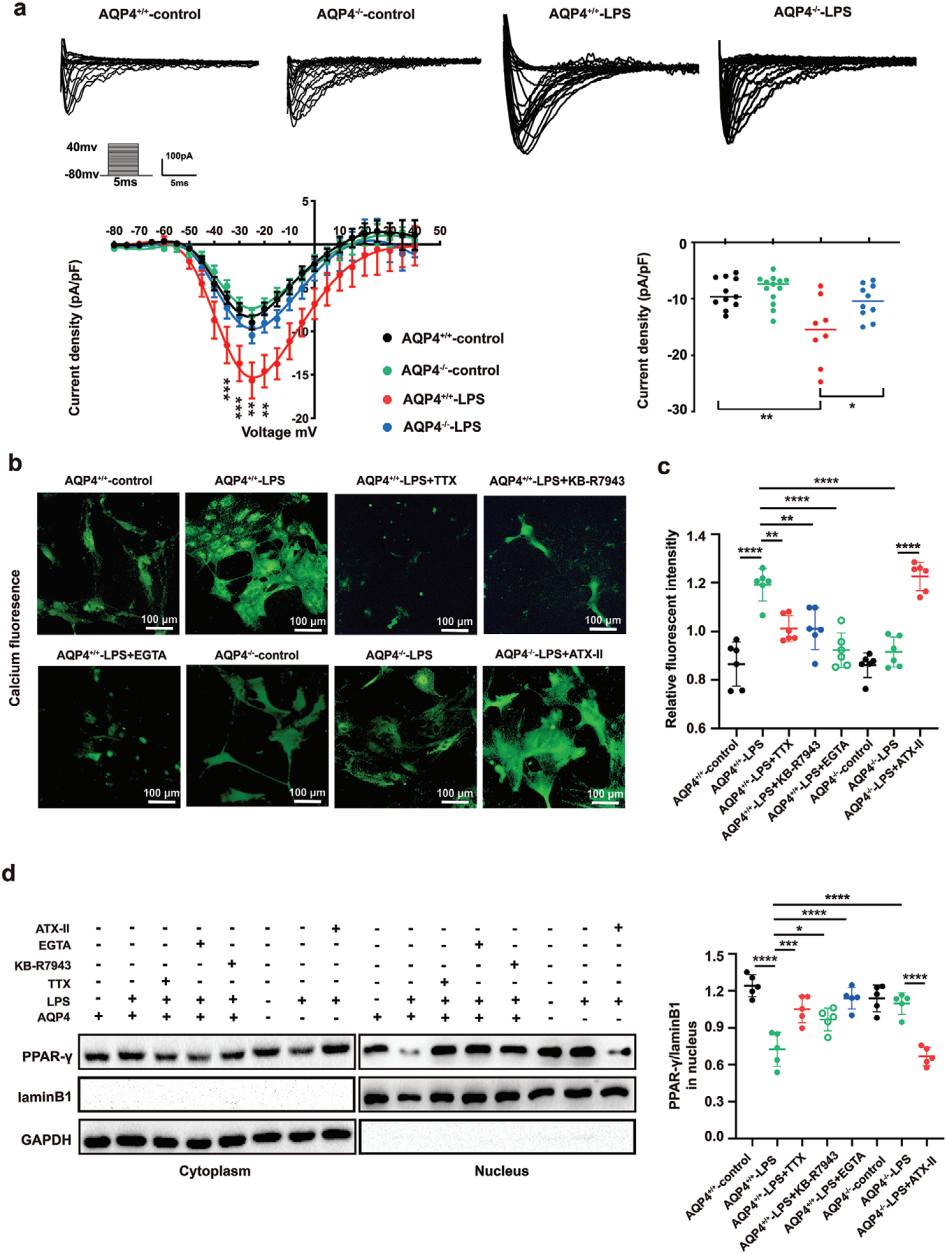
AQP4 regulates sodium‐calcium exchange through Na_v_1.6 to inhibit LPS‐induced astrocyte PPAR‐*γ* moving into the nucleus. a) Representative families of VGSC current traces in AQP4^+/+^ and AQP4^−/−^ primary astrocytes which were stimulated by LPS or not (upper). The membrane potential was held at ‐80 mV, and the currents were elicited by 5 ms test pulses ranging from ‐80 to +40 mV in 5 mV steps. Current‐voltage relationship of VGSC currents in primary astrocytes (lower left). Mean current density of VGSC at ‐25 mV in primary astrocytes under different treatments (lower right). *n* = 8–13 cells from four independent experiments. b) Representative fluorescence images of primary astrocyte incubated with Fluo‐4 AM dye in different groups. Scale bar, 100 µm. c) Measurement of Fluo‐4 AM fluorescence intensity by microplate reader after AQP4^+/+^ and AQP4^−/−^ primary astrocytes were treated with TTX, KB‐R7943 or EGTA, ATX II followed by LPS challenge or not. *n* = 6. d) Representative protein bands of PPAR‐*γ* in the nucleus after AQP4^+/+^ and AQP4^−/−^ primary astrocytes treated with TTX, KB‐R7943, EGTA, or ATX II followed by LPS challenge or not (*left*). Quantitative analysis for PPAR‐*γ*/laminB1 ratio of relative protein expression in nucleus of all groups, *n* = 5. (right). Data are presented as mean ± SD. a) ** *p* < 0.01, **** *p* < 0.0001, two‐way ANOVA with Tukey's post hoc test; c,d) * *p* < 0.05, *** *p* < 0.001, **** *p* < 0.0001, one‐way ANOVA with Tukey's post hoc test.

Previously, we have found that Na_v_1.6 produces a persistent sodium current which in turn can also drive a reverse Na^+^/Ca^2+^ exchange action to import harmful levels of calcium ions into the cytoplasm.^[^ ^30^, ^46^ ^]^ Store‐operated Ca^2+^ entry (SOCE) channels causing calcium ions import has been implicated in the regulation of many transcription factors and regulatory proteins activation, including PPAR‐*γ*.^[^ ^47^, ^48^ ^]^ The sodium currents of astrocytes were observed using whole‐cell patch clamp recording. By applying depolarizing pulses from ‐80 mV to +40 mV at 5 mV steps for 5 ms astrocyte cells were separated for experiments. Currents were normalized to membrane capacitance to calculate current densities (pA/pF) thus eliminating the influence of cell size. **Figure** **9**a shows that after being stimulated with LPS for 24 h, the current density of VGSCs of AQP4^+/+^‐LPS rose up from ‐8.997 to ‐15.75 pA pF^‐1^. Interestingly, the current density of VGSCs of AQP4^−/−^‐LPS was ‐10.46 pA pF^‐1^ which was a significant decrease compared with AQP4^+/+^‐LPS. The results showed that AQP4 knockout did not increase the sodium influx of astrocytes induced by LPS. To investigate whether AQP4 affected LPS‐induced [Ca^2+^]_i_ (intracellular calcium) levels in astrocytes, we looked into the change in Ca^2+^ levels caused by LPS treatment using an intracellular calcium indicator known as Fluo‐4 AM. The data showed that the [Ca^2+^]_i_ increased following LPS stimulation, and AQP4 knockout had attenuated this rise in [Ca^2+^]_i_ of astrocytes (Figure 9b,c). Furthermore, it was also shown that Ca^2+^ chelator EGTA, NCX (Na^+^/Ca^2+^ exchanger) inhibitor KB‐R7943 and VGSC inhibitor TTX had the same effect as in AQP4 knockout, decreasing LPS‐induced elevation of [Ca^2+^]_i_ in astrocytes. However, sodium channel activator (ATX II) mitigated the effect of AQP4 knockout on LPS‐induced primary astrocyte [Ca^2+^]_i_ elevation (Figure 9c). These results suggested that a rise in primary astrocyte [Ca^2+^]_i_ stimulated by LPS was dependent on Na^+^ influx, AQP4 knockout decreased [Ca^2+^]_i_ elevation by inhibiting the sodium channel and probably the consequent Na^+^/Ca^2+^ exchange. To further investigate whether the effect of AQP4 knockout on Na^+^ influx and [Ca^2+^]_i_ was having any relation to the PPAR‐*γ* transportation to the nucleus of astrocytes, expression of nuclear PPAR‐*γ* was measured. We have shown that AQP4 knockout can reduce LPS‐induced astrocyte activation through the activation of PPAR‐*γ*‑dependent autophagy. Consistently, LPS‐induced inhibition of PPAR‐*γ* transportation to the nucleus was activated by AQP4 knockout, TTX, KB‐R7943 or EGTA, however ATX II mitigated the effect of AQP4 knockout (Figure 9d). These results conclusively show that AQP4 knockout causes a considerable reduction in LPS induced sodium channel activation which in turn influences the Na^+^/Ca^2+^ exchange mechanism, thus attenuating PPAR‐*γ* inhibition, in the astrocytes, thereby promoting an enhanced anti‐inflammatory reaction.

### 2.8 AQP4 Knockout Activate PPAR‐*γ* to Alleviate Neuronal Injury via Na_v_1.6 Activation

**Figure 10:**
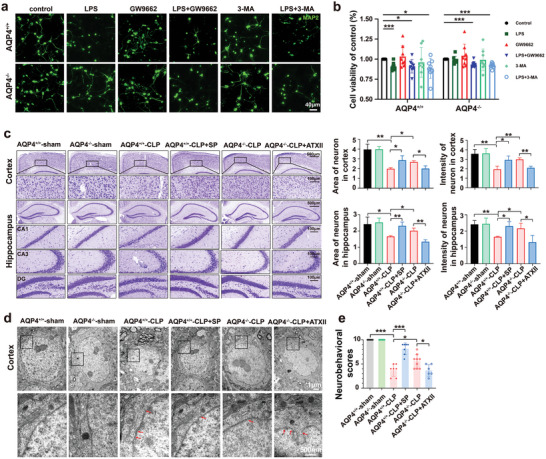
AQP4 knockout antagonized PPAR‐*γ* to alleviate neuronal injury via Na_v_1.6 activation. a) Primary neurons were immunostained with MAP2 (green). The neurons were stimulated with cell culture media of AQP4^+/+^ and AQP4^−/−^ primary astrocytes were treated with GW9662, 3‐MA followed by LPS challenge or not. scale bar, 40 µm. b) CCK8 assay was used to detect the neuron viability of each group. Survival rate = (mean absorbance of experimental group/mean absorbance of control group) × 100%. *n* = 8. c) Representative images of Nissl‐stained sections of cortex and hippocampus from different groups. Scale bar, 500 µm, 100 µm. Right panel, quantification of area and intensity of neuron in the mice cortex and hippocampus among different groups. *n* = 3 mice for each group. d) Cortical neurons of the six treatment groups, visualized by TEM. TEM analysis showed nuclear pyknosis and nuclear membrane rupture (red arrows) in AQP4^+/+^‐CLP, AQP4^+/+^‐CLP+SP, AQP4^−/−^‐CLP, AQP4^−/−^‐CLP+ATX II. Scale bar, 1 µm, 500 nm. e) The neurobehavioral scores of different group mice. *n* = 6–9 mice for each group. Data are presented as mean ± SD. * *p* < 0.05, ** *p* < 0.01, *** *p* < 0.001, one‐way ANOVA with Tukey's post hoc test.

**Figure 11:**
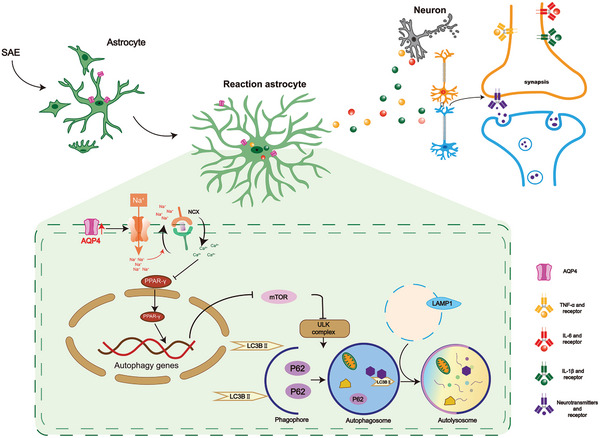
AQP4 deletion activate PPAR‐*γ* to alleviate neuronal injury via Na_v_1.6 activation in SAE. The proposed mechanism flowchart depicting interference of AQP4's effect on the progression of SAE. Sepsis can accelerate the formation of the AQP4‐Na_v_1.6 complex in astrocyte and then causes a rapid influx of Na^+^ through VGSC. The increase of Na^+^ influx could cause an increase in intracellular calcium concentration by activating the reverse mode of the Na^+^/Ca^2+^ exchanger. Ca^2+^ overload inhibits PPAR‐*γ*/mTOR‑dependent autophagy and activates inflammation response in astrocyte, which resulting in neuron injury.

Astrocyte activation releases inflammatory factors to further damage neurons, which is one of the main causes of neuron injury during neuroinflammation. To observe the effect of AQP4 knockout on the neuron injury, astrocyte conditioned medium was added to the primary culture neuron. The results showed that LPS‐induced neuron viability decrease was alleviated by AQP4 knockout, however GW9662 or 3‐MA mitigated the effect of AQP4 knockout (**Figure** **10**a,b). Next, the AQP4^+/+^‐CLP mice were administrated with scorpion venom heat‐resistant synthesized peptide [SVHRSP, patented reagent (No. ZL201610645111.7) which is one of Na_v_1.6 inhibitor from our laboratory^[^ ^30^ ^]^ and AQP4^−/−^‐CLP mice were administrated with ATX II (sodium channel activator). Cresyl violet staining was performed to evaluate neuronal death in the CLP model. We found that the neuron bodies were much larger, the color was shallower, and specifically, the area and intensity of neurons were significantly greater in the AQP4^+/+^‐CLP+SP (i.e.: SVHRSP) mice and AQP4^−/−^‐CLP mice than in the AQP4^+/+^ mice, whereas, ATX II mitigated the effect of AQP4 knockout (Figure 10c). Notably, cell nuclei pyknosis and nuclear membrane rupture in the AQP4^+/+^‐CLP+SP mice and AQP4^−/−^‐CLP mice were less obvious than that in the AQP4^+/+^‐CLP mice and AQP4^−/−^‐CLP+ATX II mice, as detected by electron microscopy (Figure 10d). AQP4 knockout and SVHRSP also increased the neurological score of septic mice, collectively, ATX II mitigated the effect of AQP4 knockout (Figure 10e). These results suggest that AQP4 knockout activates PPAR‐*γ* to alleviate neuronal injury via Na_v_1.6 inhibition. The proposed mechanisms of AQP4 deletion to alleviate neuronal injury have been summarized in **Figure** **11**.

## 3 Discussion

Some reports have demonstrated AQP4 expression to be up‐regulated during aggravated brain injury due to sepsis and brain inflammation.^[^ ^19^ ^]^ Consistent with the results, the AQP4 expression was increased in both SAE patient's peripheral blood along with the cortex and hippocampus of septic mice brain on day 1 and day 7. APACHE II increment was associated with an unfavorable patient prognosis outcome,^[^ ^49^ ^]^ relatively we also found that AQP4 was better at identifying SAE and was positively associated with APACHE II score. SAE has a significant association with a high rate of mortality and morbidity worldwide. In SAE existence of a relationship between long‐term cognitive disorders and brain lesions has been reported a variety of studies.^[^ ^10^, ^50^, ^51^ ^]^ We found that the AQP4 knockout mice exhibited remarkably improved clinically relevant indices including survival, neurological status along with SAE. Electroencephalogram (EEG) recordings with abnormal findings represent a mental dysfunction in sepsis and it may show an increased delta and decreased alpha activity even if the neurological signs are normal.^[^ ^52^, ^53^ ^]^ Certain reports have shown that in septic patients, the EEG data showed progressive slowing of brain activity with the increasing severity of SAE.^[^ ^34^ ^]^ AQP4 knockout revised the increase of delta and decrease of alpha wave activity in septic mice in our experiment. Previous studies found that neuronal injury decreased neuronal excitability and excitatory currents, reduced network activity and frequency of action potentials (Aps) in mice after brain injury.^[^ ^54^ ^]^ We also found that AQP4 deletion can also improve the neuronal excitability induced by CLP in mice using brain patch clamp. Cognitive impairment is associated strongly with synaptic loss and its functional abnormalities.^[^ ^55^, ^56^ ^]^ The result of Morris water maze test showed that AQP4 knockout alleviated the cognitive dysfunctions. Thus, we probed whether the AQP4 deletion could prevent synaptic plasticity defects or functional inabilities. LTP was used to evaluate the synaptic functional plasticity, electron microscopy and Golgi staining were used to evaluate the synaptic morphological plasticity. These results demonstrated that AQP4 knock out ameliorated cognitive dysfunction and improves synaptic plasticity of CLP‐induced sepsis in mice.

As our results, AQP4 knockout did not induce cognitive impairment in normal mice, it suggests that AQP4 is not pivotal in physiological condition, but in the SAE pathological condition. This means increase of AQP4 is stress‐related reaction. Considering that AQP4 null mice were found to have normal intracranial pressure and only slightly increased total brain water content.^[^ ^57^, ^58^ ^]^ Thus, AQP4 may not be crucial for relatively slow water movements in and out of the brain that take place under physiological conditions, as these can occur through AQP4‐independent pathways. CNS disorders (such as cerebral damage, cerebral tumors, and hydrocephalus) exhibit the rates of water flow of the cerebrum rises significantly, leading to an heightened intracranial pressure due to overload water accumulation in intracranial compartments. Increased intracranial pressure is harmful, as it causes brain ischemia, herniation ultimately causing brain death.^[^ ^59^, ^60^ ^]^ AQP4 thus facilitates water transportation of the brain related to these disorders. In our study, increase of AQP4 expression is stress‐related reaction in mice with CLP, AQP4 knockout could reverse the pathological change.

Astrocytes activation due to sepsis has been supported by growing body of evidence along with a production of inflammatory cytokines in substantial amounts.^[^ ^7^, ^12^ ^]^ In addition, post‐onset of sepsis astrocyte‐mediated neuroinflammation plays a vital role in the development of long‐term cognitive functional inability.^[^ ^39^ ^]^ The present experiments demonstrated CLP induces the activation of astrocytes and also production of proinflammatory cytokines. Thus, we might conclude, supported by the evidence, that astrocytes‐mediated neuroinflammation plays a critical role in SAE.

Autophagy is involved in the pathogenesis of SAE, but the specific role and mechanism remain to be determined. Autophagy activation leads to LC3‐I in the cytoplasm bindings with phosphatidylethanolamine and then transformed into membrane‐bound LC3‐II by P62/SQSTM1.^[^ ^61^ ^]^ Studies have shown that autophagy is significantly inhibited in the septic mice brain, and this inhibition can lead to increased production of pro‐inflammatory cytokines, which further aggravate the neuronal injury and cognitive dysfunction in the cortex and hippocampus of mice.^[^ ^62^, ^63^, ^64^ ^]^ The mechanism may be that damaged or abnormal organelles cannot be effectively cleared when autophagy inhibition, these damaged or abnormal organelles activated inflammatory complexes promote the secretion of inflammatory factors and the production of reactive oxygen species, leading to neuronal destruction.^[^ ^61^ ^]^ However, other studies have found that the activation of autophagy aggravated neuronal damage in septic mice brain.^[^ ^65^ ^]^ This may be related to the difference of observation time and severity of SAE. Studies also showed that autophagy was significantly inhibited after a brief increase in the septic mice brain.^[^ ^66^ ^]^ Our study shows that sepsis induces the production of double‐membrane autophagosomes in lesser numbers, increase in free p62, and decreased level of LC3B‐II in mouse brain, showing deregulation of autophagy in the mouse brain. An increasing level of autophagy may help mitigate sepsis‐induced brain injury was one of the conclusions in our present study. However, a balance is needed to be maintained as excessive autophagy can also cause harm to cellular homeostasis.^[^ ^63^ ^]^ Further research is required to ascertain as to which level of autophagy gives the best therapeutic effect. Recent studies have also found that inhibition astrocytic autophagy can lead to the activation of astrocytes and increased release of inflammatory factors, which is the main cause of neuronal injury,^[^ ^14^ ^]^ however upregulation astrocytic autophagy can improve neuronal activity, reduce neuronal apoptosis and improve neural function.^[^ ^15^ ^]^ AQP4 are mainly expressed at astrocytes in CNS, so we further observe the astrocyte autophagy. Interestingly, AQP4 knockout promoted astrocytic autophagy, particularly resulting in an increased level of colocalization of LC3B with GFAP, meanwhile the intensity of GFAP was reduced in septic mice. These results suggest that AQP4 knockout can promote astrocyte autophagy in SAE, thereby reducing astrocyte activation.

Recent studies show that PPAR‐*γ*/mTOR pathway may trigger autophagy^[^ ^42^, ^43^, ^44^ ^]^ and subsequent astrocyte‐mediated neuroinflammation in sepsis. Meanwhile AQP4 knockout can activate the expression of PPAR‐*γ* and decrease the release of inflammatory factors in the brain.^[^ ^45^ ^]^ AQP4‐mediated dysregulation of autophagy may also attribute to the inhibition of PPAR‐*γ*/mTOR pathway in sepsis and was suggested by our experimental results. We found that AQP4 deficiency restores PPAR‐*γ* moving into the cell nucleus, decreases the phosphorylation of p‐mTOR, and then enhances downstream activation of ULK1 and LAMP1 in cortex and hippocampus in vivo and in astrocyte cultures. PPAR‐*γ* antagonist GW9662 partially reversed these effects induced by AQP4 deletion. In acute brain injury mice model, AQP4 knockout shows a reduction in inflammatory response and a decreased activation of astrocytes.^[^ ^67^ ^]^ Similarly, data from our results suggest also that deficiency of AQP4 inhibits astrocyte‐induced inflammation, restoring astrocyte autophagy function and also autophagy inhibitor 3‐MA can counteract the effect of AQP4 deficiency. These results suggesting AQP4 deletion activates PPAR‐*γ*/mTOR‑dependent autophagy and inhibits inflammation response in vivo and in vitro.

Numerous reports have shown that Ca^2+^, as an important intracellular second messenger, can affect cell signals, including PPAR‐*γ* mediated related signaling pathways.^[^ ^68^ ^]^ Meanwhile, the membrane protein Na_v_1.6, expressed in astrocytes, plays a predominant role in modulating intracellular Ca^2+^ and neuroinflammatory response.^[^ ^22^ ^]^ Our CoIP experiments showed that astrocytic AQP4 interacted with Na_v_1.6 and LPS treatment can accelerate the formation of the AQP4‐Na_v_1.6 complex. Here, we demonstrated that Na_v_1.6 was upregulated in mice with CLP or astrocyte with LPS treatment in vitro, which in turn was suppressed by AQP4 knockout. Unique biophysical properties of Nav1.6 is characterized by persistent sodium current (INaP) and resurgent currents. INap described in many brain disorders, plays an vital pathophysiological role.^[^ ^69^ ^]^ Indeed, a sustained Na^+^ influx through the Na^+^ channel can induce calcium‐related injuries by the reverse mode of the Na^+^/Ca^2+^ exchanger.^[^ ^70^ ^]^ Thus it appears that Na_v_1.6 channel must have a crucial role and may significantly contribute to the pathophysiology of nervous system injury via INap.^[^ ^71^, ^72^ ^]^ Studies show that LPS induced stimulation of astrocyte causes a rapid influx of Na^+^ through VGSC along with Ca^2+^ overload and the release of pro‐inflammatory cytokines also including a series of inflammatory signaling events being triggered,^[^ ^46^, ^70^ ^]^ AQP4 knockout can reduce Ca^2+^ overload in astrocytes.^[^ ^73^ ^]^ Our findings are consistent with these works, LPS increased astrocytic sodium current, which in turn can also drive reverse Na^+^/Ca^2+^ exchange action to import harmful levels of Ca^2+^ into the astrocyte cytoplasm as indicated by intracellular Fluo‐4 AM fluorescence. AQP4 deficiency, as TTX, KB‐R7943, and EGTA, dramatically alleviated the effect of LPS‐induced astrocytic calcium overload. However, sodium channel activator (ATX II) mitigated the effect knockout AQP4 on LPS‐induced primary astrocyte [Ca^2+^]i elevation. Our results suggest that an increase in [Ca^2+^]i elicited by LPS within astrocyte was Na^+^ influx dependent, and AQP4 knockout reduced [Ca^2+^]i increment by inhibiting sodium channel and probably the consequent Na^+^/Ca^2+^ exchange. The increase of intracellular Ca^2+^ can activate many transcription factors and regulatory proteins, including PPAR‐*γ*.^[^ ^48^, ^74^ ^]^ Our experiment indicated that in AQP4 mediated inhibition of PPAR‐*γ*‑dependent astrocyte autophagy, Na^+^ influx and an elevated intracellular Ca^2+^ level were critical, as TTX, KB‐R7943 and EGTA dramatically suppressed LPS‐induced inhibition of PPAR‐*γ* pathway. Notably, sodium channel activator ATX II significantly inhibited the effect of AQP4 knockout on PPAR‐*γ* activation, suggesting that Na_v_1.6 is the target site of AQP4 for inhibiting PPAR‐*γ* related autophagy. These results conclusively show that AQP4 knockout causes a considerable reduction in LPS induced sodium channel activation which in turn influences the Na^+^/Ca^2 +^ exchange mechanism, thus attenuating PPAR‐*γ* inhibition, in the astrocytes, thereby promoting an enhanced anti‐inflammatory reaction.

Studies have shown that an enhanced viability of neurons and a decrease in neuronal apoptosis can be achieved by induction of autophagy flux in astrocytes.^[^ ^15^, ^61^ ^]^ We have confirmed that in a conditioned medium of astrocytes pretreated with PPAR‐*γ* antagonist GW9662 or autophagy inhibitor 3‐MA could mitigate the effect of AQP4 knockout on LPS‐induced primary astrocytes autophagy, activation and proinflammatory cytokine release. We have conclusively shown through our experimental data that neuronal rescue during recovery from LPS can be linked to AQP4 knockout. However, the conditioned medium of astrocyte pretreated with GW9662 or 3‐MA alleviated the effect of AQP4 deletion on primary neuron. We got similar result in CCK8 assay to detect the neuron viability of each group in Figure 10b. Therefore, we speculate that AQP4 knockout enhanced autophagy to alleviate neuronal injury via PPAR‐*γ* activation. SVHRSP, down‐regulating Na_v_1.6, and AQP4 knockout alleviated the neuron injury in septic mice brain, collectively, ATX II mitigated the effect of AQP4 deficiency. These results indicated that Na_v_1.6 activation is necessary for AQP4‐mediated neuron injury in vivo and in vitro.

## 4 Conclusions

In conclusion, AQP4 aggravates sepsis‐induced neuronal injury and cognitive dysfunction by inhibiting PPAR‐*γ*/mTOR‑dependent autophagy and activating inflammatory response in astrocytes. The underlying mechanisms involve increased Na^+^ influx via Na_v_1.6, which leads to intracellular Ca^2+^ overload in astrocytes. The study provides new insights into pathological mechanisms of sepsis‐induced neuronal injury and potential new drug target to treat SAE.

## 5 Experimental Section

### Animal

Male CD1 mice which were divided into AQP4 wild‐type (AQP4^+/+)^ and AQP4 knockout (AQP4^−/−^) mice (8‐10 weeks old, 20–25 g) were obtained from the Specific Pathogen Free (SPF) Model Animal Center of Dalian Medical University and were randomly grouped and housed at a humidity‐controlled along with the constant temperature‐maintained animal box. The AQP4 knockout mice were established by Ma et al.^[^ ^31^ ^]^ Animal studies are reported in compliance with the ARRIVE guidelines.^[^ ^75^, ^76^ ^]^ The animals were allowed to acclimatize for at least 1 week prior to the start of experiments.

All animal experiments were carried out according to the Institutional Animal Care and Use Committee guidelines of the NIH, USA (NIH publication no. 86‐23, revised 1987), and approved by the Institutional Ethics Committee of Dalian Medical University. All experiments were designed to minimize the number of animals used and animal suffering.

### Human‐Derived Serum Collection

Samples from the peripheral blood from thirty‐three SAE patients (conformed to the Sepsis3.0 diagnostic criteria and sepsis‐associated encephalopathy diagnostic criteria) and 27 sepsis patients (conformed to the Sepsis3.0 diagnostic criteria but not conformed sepsis associated encephalopathy diagnostic criteria) and 20 age‐matched control subjects were analyzed by ELISA. The APACHE II (acute physiology and chronic health evaluation II) score of each group was recorded to reflect the severity of patient's disease and the SOFA (sequential organ failure assessment) score was used to assess the severity of sepsis. All samples were obtained from August 2018 to November 2020, sepsis patients and sepsis‐associated encephalopathy patients admitted to the Intensive Care Department of the Second Hospital of Dalian Medical University, as well as healthy examination subjects in the Health Examination Center. Diagnosis of sepsis‐associated encephalopathy was carried out by an accredited physician, only after all the patients have given their informed consent in accordance with the hospital, where the samples were extracted.

### Enzyme Linked Immunosorbent Assay (ELISA)

Blood collection was done from the healthy control and patients. The coagulated blood was centrifuged at 3000 revolutions per minute for 20 minutes and the supernatant liquid was collected as serum. Cell culture media was collected and centrifuged to remove precipitation. The concentration of AQP4, S100*β*, NSE, TNF‐*α*, IL‐1*β*, IL‐6 extracts in serum and cell culture media was measured by using ELISA kit.

### Genotyping of AQP4^−/−^ Mice

DNA from tails were extracted in lysis buffer (5 × 10^‐3^ m EDTA: 5 × 10^‐3^ m NaOH: H_2_O = 1:1:8) 40 min at 99 °C in PCR machine. PCR was done by using 0.1–0.7 µg of DNA (1 µL) for each sample, in a final volume of 20 µL. The primers used for genotyping (Life Technologies, Thermo Fisher Scientific‐CN, Shanghai, China) are AQP4‐sense primer 5’‐ACCATAAACTGGG GTGGCTCAG‐3’, AQP4‐antisense primer 5’‐TAGAGGATGCCGGCTCCAATGA‐3’, AQP4‐neo primer 5’‐CACCGCTGAATATGCATAAGGCA‐3’. The conditions for PCR were conducted as previously reported.^[^ ^31^ ^]^ AQP4^+/+^ and AQP4^−/−^ bands were detected at 240 bp and 320 bp, respectively, compared with the standard DNA ladder. BIO‐RAD (Hercules, CA, USA) gel analysis software was used to detect band signals.

### Establishment of Cecal Ligation and Puncture (CLP) Model

Sepsis‐associated encephalopathy was induced through the cecal ligation and puncture (CLP) introduced by Daniel Rittirsch with slight modifications.^[^ ^77^ ^]^ Isoflurane inhalation was used to anesthetize the animals. The procedure was performed on animal heating pads to maintain the mice body temperature at 37 °C. Approximately 1 cm wound was dissected in the midline of the abdomen of the mouse, after location and exposure, the cecum was ligated with surgical suture. A single cecum puncture was performed with a 22G sterile needle and the cecum was gently compressed to leak a droplet of feces. Then the cecum is carefully relocated into the abdominal cavity after being sutured. Post‐surgery every animal was given resuscitation fluid (37 °C, 0.9% NaCl, 50 mL kg^‐1^, s.c.).

### Neurobehavioral Scores

Neurobehavioral scores were used to assess symptoms consistent with septic‐associated encephalopathy in mice. The health status of experimental mice was scored with the following five signs: corneal reflex, auricle reflex, righting reflex, tail flailing reflex, and escape reflex.

### Electroencephalogram (EEG)

EEG was used to record the electrical activity of unrestrained mice. Stereotaxic apparatus was used to fix the mice post‐anesthesia a, and electrodes were implanted into the cerebral cortex (post‐bregma 2.3 mm, lateral sagittal suture 2.1 mm, ventral dura 2 mm). The signals of EEG were digitally processed using AD (Lab Chart Software, AD Instruments).

### Sodium Dodecyl Sulfate‐Polyacrylamide Gel Electrophoresis (SDS‐PAGE) and Western Blot Analysis

SDS‐PAGE was conducted as previously reported with modifications.^[^ ^78^ ^]^ Brain samples were homogenized in RIPA buffer and protease inhibitor cocktail. Cytoplasmic and nuclear fractions were prepared from primary astrocytes which were treated with different drug and different groups of mice brain using Minute Cytoplasmic & Nuclear Extraction Kits (SC‐003, Invent Biotechnologies, USA). The primary antibodies used included AQP4 (rabbit, 1:1000, Abcam, ab46182), AQP4 (rabbit, 1:1000, Alomone, AQP‐004), and anti‐*β*‐actin (mouse, 1:2000, Cell Signaling Technology, 3700s), GFAP (rabbit, 1:1000, Millipore, Mab360), LC3B(rabbit, 1:1000, Cell Signaling Technology, 3868s), p62 (rabbit, 1:1000, Sigma, p0067), p‐mTOR (mouse, 1:1000, Cell Signaling Technology, 5536s), mTOR (mouse, 1:1000, Cell Signaling Technology, 2983s), p‐ULK1 (mouse, 1:1000, Cell Signaling Technology, 14202), ULK1 (mouse, 1:1000, Cell Signaling Technology, 8054), LAMP1 (mouse, 1:1000, Invitrogen,14‐1071‐82), PPAR‐*γ* (rabbit, 1:1000, Cell Signaling Technology, 2443s), GAPDH (mouse, 1:2000, Abcam, ab9484), LaminB1 (mouse, 1:1000, Abcam, ab220797), IgG (rabbit, 1:1000, Proteintech, 2729p), Na_v_1.6 (mouse, 1:1000, Abcam, ab65166), Na_v_1.6 (rabbit, 1:200, Sigma, WH0006334M4), pERK1/2 (rabbit, 1:1000, Cell Signaling Technology, 3179S), pJNK (mouse, 1:2000, Cell Signaling Technology, 9255S), p‐P38 (mouse, 1:2000, Cell Signaling Technology, 9216S), pAKT (rabbit, 1:2000, Cell Signaling Technology, 4060S), pGSK3*β* (rabbit, 1:1000, Cell Signaling Technology, 9323S) and the sample‐loaded membranes were incubated overnight at 4 degree and then post 8–12 h of incubation treated with secondary antibody, goat anti‐rabbit (1:5000, Thermo, A16104) and goat anti‐mouse IgG (1:5000, Thermo, 31430), along with electrochemiluminescence (ECL, Millipore) reagent. BIO‐RAD (Hercules, CA, USA) gel analysis software was used to detect band signals.

### Electrophysiology and Recording

The electrophysiology was conducted as per past reports. Each group of animals was sacrificed and their brain tissue extracted to be placed in ice‐cold oxygenated artificial cerebrospinal fluid (ACSF) with 5% CO_2_/95% O_2_ mixture.^[^ ^78^ ^]^ Coronal hippocampal slices (300 µm) were prepared from the resected brains of mice using an oscillating microtome (Leica vibratome VT‐1200; Leica Biosystems, Nussloch, Germany). Poststimulating CA3 neurons the field excitatory postsynaptic potentials (fEPSPs) in CA1 neurons were recorded. High frequency was applied to induce long‐term potentiation (LTP). LTP amplitude was quantified as the percentage change (40%) in the slope of fEPSP within 60 min after LTP induction. Paired impulse facilitation (PPF), was evaluated at stimulus intervals (ISI) of 25, 50, 75, 100, 125, 150, and 200 ms. The paired‐pulse ratio was determined as the ratio between the second pulse‐evoked of fEPSP and the first one. The current clamp was applied to record whole cell spontaneous and evoked action potentials. The whole cell block was formed by adding GABA receptor antagonist (Picrotoxin, 100 × 10^‐6^ m) into the artificial cerebrospinal fluid. The clamping potential was ‐80 mV, and the spontaneous excitatory postsynaptic current (sEPSC) was recorded in voltage clamp mode. The electrophysiological data were acquired with an Axon multiclamp 700 B amplifier, filtered at 0.1‐5 kHz, and digitized at 10 kHz, and the slope and peak amplitude of fEPSP were measured and analyzed offline using pClamp10.3 software (Molecular Devices Corp, USA).

### Morris Water Maze Assessment

The Morris water maze was conducted as reported previously.^[^ ^78^ ^]^ Animal training was done at water temperature maintained within23 ± 0.5 °C. Mice from different groups received acquisition training every 5 d with all the animals taking part in four different quadrants of training per day. In the target quadrant, the target platform, which is 10 cm in diameter, is 1 cm below the surface of the water. In the test phase, removed the platform and detected the crossing target quadrant times of each mouse. A digital video camera connected to a computer‐controlled system (Ethovision 2.0, Noldus, Wageningen, Netherlands) was used to collect mouse activity. All tests were each blind to the treatment schedule.

### Golgi Staining for Dendritic Spines

The Golgi staining was conducted as reported previously.^[^ ^78^ ^]^ Dendritic spines observed in brain tissue of each group of mice by Golgi‐Cox staining were performed using the FD Rapid Golgi Stain Kit (FD Neuro Technologies, Columbia, MD, USA). Hippocampal neuronal pictures were taken by Pannoramic MIDI Scanner (3DHistech Ltd., Budapest, Hungary).

### Immunofluorescence

Immunofluorescent staining was conducted as previously reported by Jiang et al.^[^ ^78^ ^]^ Briefly, brain samples were fixed overnight in 4% paraformaldehyde and then cryosectioned at 10 µm depth and subjected to immunofluorescence staining. Astrocytes and neuron are pretreated with drugs. And then primary astrocytes or neurons are removed from the cell incubator. After washing, cells were fixed in 4% paraformaldehyde, permeabilized in 0.3% TX‐100, and blocked in 2% bovine serum albumin (BSA). The cells were incubated with primary antibodies, including mouse anti‐GFAP (1:400, Chemicon MAB360), rabbit anti‐GFAP (1:400, DAKO, Z0334), rabbit anti‐AQP4 (1:400, Sigma, A5971), rabbit anti‐LC3B (1:400, Cell Signaling Technology, 3868S) and mouse anti‐Na_v_1.6 (1:200, Abcam, ab65166) at 4 °C overnight. The cells were then incubated with the secondary antibodies, Alexa‐594‐conjugated donkey anti‐mouse (1:400, Invitrogen, A‐21203), Alexa‐488‐conjugated donkey anti‐mouse (Invitrogen, A‐21202, 1:400), Alexa‐488‐conjugated goat anti‐rabbit (1:400, Invitrogen, A‐11008) and Alexa‐594‐conjugated goat anti‐rabbit (1:400, Invitrogen, A‐11012) and 4',6‐diamidino‐2‐phenylindole (DAPI) stain were incubated for 1 h at room temperature (RT). All images were collected with confocal microscope (Leica TCS SP8 3X). The positive fluorescence staining were calculated via random selection using ImageJ software (NIH) and Image‐Pro Plus 6.0.

### RT‐PCR

The RT‐PCR was conducted as reported previously.^[^ ^30^ ^]^ Total RNA from brain tissues was extracted with Trizol reagent. One microgram of total RNA was reverse‐transcribed into cDNA using TransScript One‐Step gDNA Removal and cDNA Synthesis SuperMix (TransGen Biotech, Beijing, China). The primers used for IL‐6, IL‐1*β*, TNF‐*α* and GAPDH (Life Technologies, Thermo Fisher Scientific‐CN, Shanghai, China) are listed in **Table** **1**.

**Table 1:** Primer sequences used

Gene	Forward primer (5’‐3’)	Reverse primer (5’‐3’)
GAPDH	CACTGGCATGGCCTTCCGT	CTTACTCCTTGGAGGCCAT
Na_v_1.6 IL‐6 IL‐1*β* TNF‐*α*	CTCCAAGAAGCCACAGAAGC TCCATCCAGTTGCCTTCTTGG TCATTGTGGCTGTGGAGAAG CGTCAGCCGATTTGCTATCT	ATGGAGAGGATGACCACCAC CCACGATTTCCCAGAGAACATG AGGCCACAGGTATTTTGTCG CGGACTCCGCAAAGTCTAAG

### Electron Microscopy

Electron microscopy was done according to protocols in Wang et al.^[^ ^79^ ^]^ The brain ultrastructure was evaluated after 24 h post CLP. The brains were collected and maintained in 2.0% paraformaldehyde and 2.0% glutaraldehyde for 24 h at 4 °C. Samples were postfixed, washed, and then embedded in epoxy resin. Sections were stained with toluidine blue and examined with light microscopy. Ultrathin sections (60 nm) were stained with 2.0% uranyl acetate and lead citrate and examined under a JEOL electron microscope (JEM‐2000EX).

### Primary Astrocyte and Neuron Culture

Primary astrocytes were prepared as previously described^[^ ^80^ ^]^ from 1 d old mice. Briefly, ice packs were used to freeze animals and minimize pain prior to decapitation and brain tissue extraction. The brain tissues were digested with 0.125% trypsin in Dulbecco's modified eagle medium (DMEM) in a cell culture incubator for 30 min at 37 °C. DMEM containing 10% fetal bovine serum (FBS) was used to terminate the digestion process. The individual cells were seeded at a density of 1 × 10^4^ cm^‐2^ into a 24‐well culture plate (NEST Biotechnology) precoated with polylysine. After 72 h, the culture medium was changed to fresh complete medium (if primary neurons were cultured, the culture medium was replaced with neurobasal after 4 h). Cells were cultured for approximately 7 d to reach a cell growth coverage area of 90%. The plates were rotated at 260 rpm (24 h, 37 °C) to collect purified astrocytes. The cells exhibited >98% positive staining for glial fibrillary acidic protein (GFAP). Astrocytes of third‐generation were used. Primary astrocytes were pretreated with 3‐MA (Figure S6, Supporting Information) or induced by LPS (Figure S7, Supporting Information).

### Quantitative Real‐Time PCR (qRT‐PCR)

qRT‐PCR was conducted as reported previously.^[^ ^80^ ^]^ The primers used for Na_v_1.6 and GAPDH (Life Technologies, Thermo Fisher Scientific‐CN, Shanghai, China) are listed in Table 1.

### Nissl Staining

Wang et al's report was used as a reference to perform Nissl staining.^[^ ^81^ ^]^ Brain samples were perfused with sterile saline, fixed overnight in 4% phosphate‐buffered formaldehyde, and graded sucrose concentrations were used to dehydrate the sections, 10 µm depth sections were prepared for staining (Leica CM 1850, Leica Microsystems AG, Wetzlar, Germany), stained with 0.1% cresyl violet for 10 min and then gradient elution of graded ethanol concentration was used to dehydrate. Finally, the sections were immersed in dimethyl benzene for 3 min twice and neutral balsam was used to seal it.

### Determination of Intracellular Ca^2+^ Levels

Detecting of intracellular Ca^2+^ levels was conducted as reported previously.^[^ ^30^ ^]^ Briefly, the intracellular Ca^2+^ of astrocyte was detected by Fluo‐4 AM. The cells were washed 3 times with Hank's buffered salt solution (HBSS) and then incubated in a cell incubator with 2.5 × 10^‐6^ m calcium indicator Flou4‐AM for 40 min. Baseline calcium levels were assigned after the dye was removed and the cells transferred to HBSS solution. Fluorescence images were captured with confocal microscope (Leica TCS SP8 3X), and the fluorescence intensity of Fluo‐4 AM was measured with a microplate reader (Bio‐rad, Hercules, CA, USA).

### Molecular Docking Study

Molecular docking study was performed using Autodock 4.0. The crystal structure of AQP4 (PDB:3GD8) was selected for this docking simulation and the crystal structure simulation of Na_v_1.6 (SCN8A) is based on crystal structure of Na_v_1.2 (SCN2A) (PDB:4JPZ) were selected for this docking simulation. Accelrys Discovery Studio Visualizer 4.5 was used for graphic display.

### Co‐IP of AQP4 with Na_v_1.6 in LPS‐Induced Primary Astrocytes

Co‐IP of AQP4 with Na_v_1.6 in LPS‐induced primary astrocytes was done using a Co‐IP kit following the manufacturer's instructions. Briefly, primary astrocyte lysate was prepared by incubating different groups of primary astrocytes with IP Lysis/Wash Buffer (Pierce) at 4 °C for 5 min. Different groups of primary astrocyte lysate were incubated with AQP4 and Na_v_1.6 specific Mab‐conjugated which were diluted by IP Lysis/Wash Buffer (Pierce) to 500 µL overnight at 4 °C. Clean the Protein A/G Magnetic Beads (Pierce) twice with IP Lysis/Wash Buffer (Pierce), then bind the antigen sample/antibody conjugate and the Protein A/G Magnetic Beads (Pierce). Upon elution, AQP4 and Nav1.6 proteins were separated and transferred to PVDF membrane. Upon Co‐IP, AQP4 and Na_v_1.6 were detected by Western blotting using AQP4 and Nav1.6‐specific MAbs, IgG‐specific MAbs were set as negative control.

### Experimental Design and Statistical Analysis

All quantitative analyses were performed with the researcher blinded to the condition. All data were shown as the mean ± standard deviation (SD). Prism 8.0.2 (GraphPad Software) was used to produce all graphs. First, a data normality test was performed, and then statistical significance was evaluated by performing unpaired Student's t tests and Mann‐Whitney U tests or ANOVA for multiple comparisons, two‐way ANOVA analysis with Tukey's post hoc test, one‐way ANOVA analysis with Tukey's post hoc test. Significance in the cumulative survival studies was determined with the log‐rank (Mantel‐Cox) test. Spearman's rank test was used to assess the correlations for univariate analyses and linear regression for multivariate analysis. Receiver operating characteristic (ROC) curve analysis was used to assess the performance of each test. A value of *p* < 0.05 was considered statistically significant.

### Ethics Statement

Human‐derived serum was obtained with consent. Obtaining and using human serum were approved by the Ethics Committee of our institution (The Second Hospital of Dalian Medical University, Dalian, China). All animal experiments were carried out according to the Institutional Animal Care and Use Committee guidelines of the NIH, USA (NIH publication no. 86‐23, revised 1987), and approved by the Institutional Ethics Committee of Dalian Medical University. All experiments were designed to minimize the number of animals used and animal suffering.

## Conflict of Interest

The authors declare no conflict of interest.

## Author Contributions

D.‐D.Z. and Y.‐L.H. contributed equally to this work. S.L., T.‐H.M., and J.Y. contributed to the conception and design of the project. D.‐D.Z. played a major role in performing experiments and analyzing the data, as well as writing the initial draft of the manuscript. D.‐D.Z., Y.‐L.H., S.‐Y.G., N.L., X.‐W.Y., A.‐R.S., Q.W., Y.Z., Y.K., Q.‐F.L., T.Z., and W.‐F.Z. contributed to conducting the experiments and analysis of the data. A.‐P.L. modified the language. All authors read and approved the final version of the manuscript.

## Supporting information

Supporting Information

## Data Availability

Research data are not shared.

## References

[1] H.‐Y. Chung , J. Wickel , F. M. Brunkhorst , C. Geis , J. Clin. Med. 2020, 9, 703. 32150970 10.3390/jcm9030703 PMC7141293

[2] H.‐C. Tian , J.‐F. Zhou , L.i Weng , X.‐Y. Hu , J.‐M. Peng , C.‐Y. Wang , W. Jiang , X.‐P. Du , X.‐M. Xi , Y.‐Z. An , M.‐L. Duan , B. Du , Chin. Med. J. 2019, 132, 2039. 31425273 10.1097/CM9.0000000000000392 PMC6793784

[3] K. Thompson , B. Venkatesh , S. Finfer , Intern. Med. J. 2019, 49, 160. 30754087 10.1111/imj.14199

[4] P. P. Pandharipande , T. D. Girard , J. C. Jackson , A. Morandi , J. L. Thompson , B. T. Pun , N. E. Brummel , C. G. Hughes , E. E. Vasilevskis , A. K. Shintani , K. G. Moons , S. K. Geevarghese , A. Canonico , R. O. Hopkins , G. R. Bernard , R. S. Dittus , E. W. Ely , N. Engl. J. Med. 2013, 369, 1306. 24088092 10.1056/NEJMoa1301372 PMC3922401

[5] D. C. Angus , W. T. Linde‐Zwirble , J. Lidicker , G. Clermont , J. Carcillo , M. R. Pinsky , Crit. Care Med. 2001, 29, 1303. 11445675 10.1097/00003246-200107000-00002

[6] C. Ren , R.‐Q.i Yao , H. Zhang , Y.‐W. Feng , Y.‐M. Yao , J. Neuroinflammation 2020, 17, 14. 31924221 10.1186/s12974-020-1701-3 PMC6953314

[7] B. Mei , J. Li , Z. Zuo , Brain, Behav., Immun. 2021, 91, 296. 33039659 10.1016/j.bbi.2020.10.008 PMC7749843

[8] M. Cai , B. Du , Y. Si , J. Miao , J. Ge , J. Zhang , J. Song , H. Bao , Am. J. Transl. Res. 2021, 13, 7538. 34377234 PMC8340252

[9] M. Gu , X.‐L. Mei , Y.‐N. Zhao , Neurotoxic. Res. 2021, 39, 489. 10.1007/s12640-020-00270-5 32876918

[10] X.‐E. Xu , L. Liu , Y.‐C. Wang , C.‐T. Wang , Q. Zheng , Q.‐X. Liu , Z.‐F. Li , X.‐J. Bai , X.‐H. Liu , Brain, Behav., Immun. 2019, 80, 859. 31145977 10.1016/j.bbi.2019.05.038

[11] B. Haileselassie , A. U. Joshi , P. S. Minhas , R. Mukherjee , K. I. Andreasson , D. Mochly‐Rosen , J. Neuroinflammation 2020, 17, 36. 31987040 10.1186/s12974-019-1689-8 PMC6986002

[12] M. Griton , I. Dhaya , R. Nicolas , G. Raffard , O. Periot , B. Hiba , J. P. Konsman , Brain, Behav., Immun. 2020, 83, 200. 31622656 10.1016/j.bbi.2019.10.012

[13] N. J. Allen , C. Eroglu , Neuron 2017, 96, 697. 29096081 10.1016/j.neuron.2017.09.056 PMC5687890

[14] X.‐S. Wang , J. Yue , L.‐N. Hu , Z. Tian , K. Zhang , L. Yang , H.‐N. Zhang , Y.‐Y. Guo , B. Feng , H.‐Y. Liu , Y.‐M. Wu , M.‐G. Zhao , S.‐B. Liu , Glia 2020, 68, 27. 31429156 10.1002/glia.23697

[15] X. Liu , F. Tian , S. Wang , F. Wang , L. Xiong , Rejuvenation Res. 2018, 21, 405. 29125039 10.1089/rej.2017.1999

[16] T. Nakada , I. L. Kwee , Neuroscientist 2019, 25, 155. 29799313 10.1177/1073858418775027 PMC6416706

[17] M. Michels , A. V. Steckert , J. Quevedo , T. Barichello , F. Dal‐Pizzol , Intensive Care Med. Exp. 2015, 3, 30. 26515197 10.1186/s40635-015-0066-x PMC4626467

[18] W. Dai , J. Yan , G. Chen , G. Hu , X. Zhou , X. Zeng , Int. J. Mol. Med. 2018, 42, 1716. 29956748 10.3892/ijmm.2018.3749

[19] K. Rump , M. Adamzik , Cell Biosci. 2018, 8, 10. 29449936 10.1186/s13578-018-0211-9 PMC5807818

[20] C. Robba , I. A. Crippa , F. S. Taccone , Curr. Neurol. Neurosci. Rep. 2018, 18, 82. 30280261 10.1007/s11910-018-0895-6

[21] J. K. J. Diss , S. P. Fraser , M. B. A. Djamgoz , Eur. Biophys. J. 2004, 33, 180. 14963621 10.1007/s00249-004-0389-0

[22] J. A. Black , S. G. Waxman , Neuron 2013, 80, 280. 24139034 10.1016/j.neuron.2013.09.012

[23] L. W. Pappalardo , J. A. Black , S. G. Waxman , Glia 2016, 64, 1628. 26919466 10.1002/glia.22967 PMC5730353

[24] K. A. Reese , J. H. Caldwell , Glia 1999, 26, 92. 10088676 10.1002/(sici)1098-1136(199903)26:1<92::aid-glia10>3.0.co;2-4

[25] H. Zhu , W. Lin , Y. Zhao , Z. Wang , W. Lao , P. Kuang , H. Zhou , Brain Res. Bull. 2017, 132, 20. 28434994 10.1016/j.brainresbull.2017.04.008

[26] X. Qiao , T. R. Werkman , J. A. Gorter , W. J. Wadman , E. A. Van Vliet , Epilepsy Res. 2013, 106, 17. 23886654 10.1016/j.eplepsyres.2013.06.006

[27] T. Sato , M. Fujita , Y. Shimizu , H. Kanetaka , L. W. G. Chu , P. D. Côté , H. Ichikawa , Neurochem. Res. 2015, 40, 124. 25380697 10.1007/s11064-014-1475-z

[28] H. Zhu , Y. Zhao , H. Wu , N. Jiang , Z. Wang , W. Lin , J. Jin , Y. Ji , Sci. Rep. 2016, 6, 38108. 27905510 10.1038/srep38108 PMC5131488

[29] M. M. Hossain , J. Liu , J. R. Richardson , Toxicol. Sci. 2017, 155, 112. 27655349 10.1093/toxsci/kfw187 PMC6080855

[30] X. Li , X. Wu , N.a Li , D. Li , A. Sui , K. Khan , B. Ge , S. Li , S. Li , J. Zhao , Br. J. Pharmacol. 2021, 178, 3553. 33886140 10.1111/bph.15502

[31] T. Ma , B. Yang , A. Gillespie , E. J. Carlson , C. J. Epstein , A. S. Verkman , J. Clin. Invest. 1997, 100, 957. 9276712 10.1172/JCI231 PMC508270

[32] P. Denver , H. D'adamo , S. Hu , X. Zuo , C. Zhu , C. Okuma , P. Kim , D. Castro , M. R. Jones , C. Leal , M. Mekkittikul , E. Ghadishah , B. Teter , H. V. Vinters , G. M. Cole , S. A. Frautschy , Front. Physiol. 2019, 10, 1269. 31708792 10.3389/fphys.2019.01269 PMC6821690

[33] E. J. Goetzl , C. B. Peltz , M. Mustapic , D. Kapogiannis , K. Yaffe , J. Neurotrauma 2020, 37, 382. 31441374 10.1089/neu.2019.6711 PMC6964810

[34] T. E. Gofton , G. B. Young , Nat. Rev. Neurol. 2012, 8, 557. 22986430 10.1038/nrneurol.2012.183

[35] S. Sri , C.‐M. Pegasiou , C. A. Cave , K. Hough , N. Wood , D. Gomez‐Nicola , K. Deinhardt , D. Bannerman , V. H. Perry , M. Vargas‐Caballero , Acta Neuropathol. Commun. 2019, 7, 25. 30795807 10.1186/s40478-019-0670-1 PMC6387506

[36] R. A. Nicoll , Neuron 2017, 93, 281. 28103477 10.1016/j.neuron.2016.12.015

[37] P. Paoletti , C. Bellone , Q. Zhou , Nat. Rev. Neurosci. 2013, 14, 383. 23686171 10.1038/nrn3504

[38] M. Zeng , Y. Shang , Y. Araki , T. Guo , R. L. Huganir , M. Zhang , Cell 2016, 166, 1163. 27565345 10.1016/j.cell.2016.07.008 PMC5564291

[39] B. Bellaver , J. P. Dos Santos , D. T. Leffa , L. D. Bobermin , P. H. A. Roppa , I. L. Da Silva Torres , C.‐A. Gonçalves , D. O. Souza , A. Quincozes‐Santos , Mol. Neurobiol. 2018, 55, 2685. 28421541 10.1007/s12035-017-0526-2

[40] T. Shulyatnikova , A. Verkhratsky , Neurochem. Res. 2020, 45, 83. 30778837 10.1007/s11064-019-02743-2 PMC7089215

[41] Y. Dong , E. N. Benveniste , Glia 2001, 36, 180. 11596126 10.1002/glia.1107

[42] F. Dell'accio , J. Sherwood , Ann. Rheum. Dis. 2015, 74, 477. 25589512 10.1136/annrheumdis-2014-206884 PMC4345836

[43] Z. Zhang , M. Guo , S. Zhao , W. Xu , J. Shao , F. Zhang , L.i Wu , Y. Lu , S. Zheng , Biomed. Pharmacother. 2015, 74, 17. 26349958 10.1016/j.biopha.2015.06.003

[44] C. Feng , D. Li , M. Chen , L. Jiang , X. Liu , Q. Li , C. Geng , X. Sun , G. Yang , L. Zhang , X. Yao , Chem.‐Biol. Interact. 2019, 311, 108795. 31419397 10.1016/j.cbi.2019.108795

[45] F. Zhao , J. Deng , X. Xu , F. Cao , K. Lu , D. Li , X. Cheng , X. Wang , Y. Zhao , J. Neuroinflammation 2018, 15, 157. 29793504 10.1186/s12974-018-1203-8 PMC5968550

[46] L. Annunziato , G. Pignataro , G. F. Di Renzo , Pharmacol. Rev. 2004, 56, 633. 15602012 10.1124/pr.56.4.5

[47] D. Chuderland , G. Marmor , A. Shainskaya , R. Seger , J. Biol. Chem. 2008, 283, 11176. 18268018 10.1074/jbc.M709030200

[48] J. S. Wiegert , H. Bading , Cell Calcium 2011, 49, 296. 21163523 10.1016/j.ceca.2010.11.009

[49] E. J. Giamarellos‐Bourboulis , A. Norrby‐Teglund , V. Mylona , A. Savva , I. Tsangaris , I. Dimopoulou , M. Mouktaroudi , M. Raftogiannis , M. Georgitsi , A. Linnér , G. Adamis , A. Antonopoulou , E. Apostolidou , M. Chrisofos , C. Katsenos , I. Koutelidakis , K. Kotzampassi , G. Koratzanis , M. Koupetori , I. Kritselis , K. Lymberopoulou , K. Mandragos , A. Marioli , J. Sundén‐Cullberg , A. Mega , A. Prekates , C. Routsi , C. Gogos , C.‐J. Treutiger , A. Armaganidis , et al, Crit. Care 2012, 16, R149. 22873681 10.1186/cc11463 PMC3580738

[50] F. Mina , C. M. Comim , D. Dominguini , O. J. Cassol Jr. , D. M. Dall`Igna , G. K. Ferreira , M. C. Silva , L. S. Galant , E. L. Streck , J. Quevedo , F. Dal‐Pizzol , Mol. Neurobiol. 2014, 49, 1069. 24234155 10.1007/s12035-013-8581-9

[51] A. C. Calsavara , D. H. Rodrigues , A. S. Miranda , P. A. Costa , C. X. Lima , M. C. Vilela , M. A. Rachid , A. L. Teixeira , Neurotoxic. Res. 2013, 24, 103. 10.1007/s12640-012-9364-1 23224747

[52] J. X. Wilson , G. B. Young , Can. J. Neurol. Sci. 2003, 30, 98.12774948

[53] A. Semmler , S. Hermann , F. Mormann , M. Weberpals , S. A. Paxian , T. Okulla , M. Schäfers , M. P. Kummer , T. Klockgether , M. T. Heneka , J. Neuroinflammation 2008, 5, 38. 18793399 10.1186/1742-2094-5-38 PMC2553764

[54] P. A. Sawant‐Pokam , T. J. Vail , C. S. Metcalf , J. L. Maguire , T. O. Mckean , N. O. Mckean , K. C. Brennan , J. Clin. Invest. 2020, 130, 6005. 33044227 10.1172/JCI134793 PMC7598047

[55] M.‐Y. Zhang , C.‐Y.i Zheng , M.‐M. Zou , J.‐W. Zhu , Y. Zhang , J. Wang , C.‐F. Liu , Q.‐F. Li , Z.‐C. Xiao , S. Li , Q.‐H. Ma , R.‐X. Xu , Neurobiol. Aging 2014, 35, 2713. 25044076 10.1016/j.neurobiolaging.2014.06.009

[56] D.‐J. Yuan , G. Yang , W. Wu , Q.‐F. Li , D.‐E. Xu , M. Ntim , C.‐Y. Jiang , J.‐C. Liu , Y. Zhang , Y.‐Z. Wang , D.‐D. Zhu , S. Kundu , A.‐P. Li , Z.‐C. Xiao , Q.‐H. Ma , S. Li , Aging Cell 2022, 21, e13593. 35353937 10.1111/acel.13593 PMC9124306

[57] M. C. Papadopoulos , G. T. Manley , S. Krishna , A. S. Verkman , FASEB J. 2004, 18, 1291. 15208268 10.1096/fj.04-1723fje

[58] M. C. Papadopoulos , A. S. Verkman , Nat. Rev. Neurosci. 2013, 14, 265. 23481483 10.1038/nrn3468 PMC3732112

[59] A. Marmarou , J. Neurosurg. 2007, 22, 1.

[60] M. C. Papadopoulos , A. S. Verkman , Pediatr. Nephrol. 2007, 22, 778. 17347837 10.1007/s00467-006-0411-0 PMC6904420

[61] K. Sung , M. Jimenez‐Sanchez , J. Mol. Biol. 2020, 432, 2605. 31931011 10.1016/j.jmb.2019.12.041

[62] A. Manfredini , L. Constantino , M. C. Pinto , M. Michels , H. Burger , L. W. Kist , M. C. Silva , L. M. Gomes , D. Dominguini , A. Steckert , C. Simioni , M. Bogo , E. Streck , T. Barichello , J. De Quevedo , M. Singer , C. Ritter , F. Dal‐Pizzol , Clin. Sci. 2019, 133, 1993. 10.1042/CS20190351 31527095

[63] R.‐X. Zhou , Y.‐Y. Li , Y. Qu , Q. Huang , X.‐M. Sun , D.‐Z. Mu , X.‐H. Li , CNS Neurosci. Ther. 2020, 26, 177. 31612615 10.1111/cns.13229 PMC6978258

[64] X. Zhuang , Y. Yu , Y.i Jiang , S. Zhao , Y. Wang , L. Su , K. Xie , Y. Yu , Y. Lu , G. Lv , Int. Immunopharmacol. 2020, 81, 106287. 32058932 10.1016/j.intimp.2020.106287

[65] Y. Li , L.i Zhang , J. Tang , X. Yang , J. Huang , T. Zhu , F. Zhao , S. Li , X. Li , Y.i Qu , D. Mu , Brain Res. Bull. 2019, 148, 79. 30940475 10.1016/j.brainresbull.2019.03.015

[66] J. Ho , J. Yu , S. H. Wong , L. Zhang , X. Liu , W. T. Wong , C. C. H. Leung , G. Choi , M. H. T. Wang , T. Gin , M. T. V. Chan , W. K. K. Wu , Autophagy 2016, 12, 1073. 27172163 10.1080/15548627.2016.1179410 PMC4990998

[67] A. M. Fukuda , J. Badaut , J. Neuroinflammation 2012, 9, 279. 23270503 10.1186/1742-2094-9-279 PMC3552817

[68] L. Liu , N. A. Clipstone , J. Cell. Biochem. 2007, 100, 161. 16888802 10.1002/jcb.21044

[69] S. G. Waxman , Nat. Rev. Neurosci. 2006, 7, 932. 17115075 10.1038/nrn2023

[70] P. Stys , S. Waxman , B. Ransom , J. Neurosci. 1992, 12, 430. 1311030 10.1523/JNEUROSCI.12-02-00430.1992 PMC6575619

[71] M. J. Craner , Brain 2004, 127, 294. 14662515 10.1093/brain/awh032

[72] M. J. Craner , J. Newcombe , J. A. Black , C. Hartle , M. L. Cuzner , S. G. Waxman , Proc. Natl. Acad. Sci. USA 2004, 101, 8168. 15148385 10.1073/pnas.0402765101 PMC419575

[73] M. Xiao , G. Hu , CNS Neurosci. Ther. 2014, 20, 385. 24712483 10.1111/cns.12267 PMC6493026

[74] N. Heming , A. Mazeraud , F. Verdonk , F. A. Bozza , F. Chrétien , T. Sharshar , Crit. Care 2017, 21, 65. 28320461 10.1186/s13054-017-1643-z PMC5360026

[75] N. Percie Du Sert , V. Hurst , A. Ahluwalia , S. Alam , M. T. Avey , M. Baker , W. J. Browne , A. Clark , I. C. Cuthill , U. Dirnagl , M. Emerson , P. Garner , S. T. Holgate , D. W. Howells , N. A. Karp , S. E. Lazic , K. Lidster , C. J. Maccallum , M. Macleod , E. J. Pearl , O. H. Petersen , F. Rawle , P. Reynolds , K. Rooney , E. S. Sena , S. D. Silberberg , T. Steckler , H. Würbel , PLoS Biol. 2020, 18, e3000410. 32663219 10.1371/journal.pbio.3000410 PMC7360023

[76] E. Lilley , S. C. Stanford , D. E. Kendall , S. P. H. Alexander , G. Cirino , J. R. Docherty , C. H. George , P. A. Insel , A. A. Izzo , Y. Ji , R. A. Panettieri , C. G. Sobey , B. Stefanska , G. Stephens , M. Teixeira , A. Ahluwalia , Br. J. Pharmacol. 2020, 177, 3611. 32662875 10.1111/bph.15178 PMC7393193

[77] D. Rittirsch , M. S. Huber‐Lang , M. A. Flierl , P. A. Ward , Nat. Protoc. 2009, 4, 31. 19131954 10.1038/nprot.2008.214 PMC2754226

[78] R. Jiang , X.‐F. Wu , B. Wang , R.‐X. Guan , L.‐M. Lv , A.‐P. Li , L. Lei , Y. Ma , N. Li , Q.‐F. Li , Q.‐H. Ma , J. Zhao , S. Li , Alzheimer's Res. Ther. 2020, 12, 47. 32331528 10.1186/s13195-020-00616-3 PMC7181577

[79] X. Wang , X.‐L. Yang , W.‐L. Kong , M.‐L. Zeng , L. Shao , G.‐T. Jiang , J.‐J. Cheng , S. Kong , X.‐H. He , W.‐H. Liu , T.‐X. Chen , B.‐W. Peng , J. Neuroinflammation 2019, 16, 214. 31722723 10.1186/s12974-019-1618-x PMC6852893

[80] M. Ntim , Q.‐F. Li , Y. Zhang , X.‐D. Liu , N. Li , H.‐L. Sun , X. Zhang , B. Khan , B. Wang , Q. Wu , X.‐F. Wu , W. Walana , K. Khan , Q.‐H. Ma , J. Zhao , S. Li , Cereb. Cortex 2020, 30, 4617. 32219328 10.1093/cercor/bhaa064

[81] X.‐G. Wang , D.‐D. Zhu , N. Li , Y.‐L. Huang , Y.‐Z. Wang , T. Zhang , C.‐M. Wang , B. Wang , Y. Peng , B.‐Y. Ge , S. Li , J. Zhao , Neurosci. Bull. 2020, 36, 243. 31502213 10.1007/s12264-019-00425-1 PMC7056763

