# AQP4 Aggravates Cognitive Impairment in Sepsis‐Associated Encephalopathy through Inhibiting Nav1.6‐Mediated Astrocyte Autophagy

**DOI:** 10.1002/advs.202306241

**Published:** 2023-10-26

**Authors:** Dan‐Dan Zhu, Yue‐Lin Huang, Song‐Yu Guo, Na Li, Xue‐Wei Yang, Ao‐Ran Sui, Qiong Wu, Yue Zhang, Yue Kong, Qi‐Fa Li, Ting Zhang, Wen‐Fei Zheng, Ai‐Ping Li, Jian Yu, Tong‐Hui Ma, Shao Li

*Adv. Sci*. **2023**, *10*, 2205862

https://doi.org/10.1002/advs.202205862

In the originally published article there are errors in Figure 3 and 10. The correct figures are reproduced below. These errors do not affect the results or conclusions of this article. The authors apologize for any inconvenience this may have caused.

**Figure 3:**
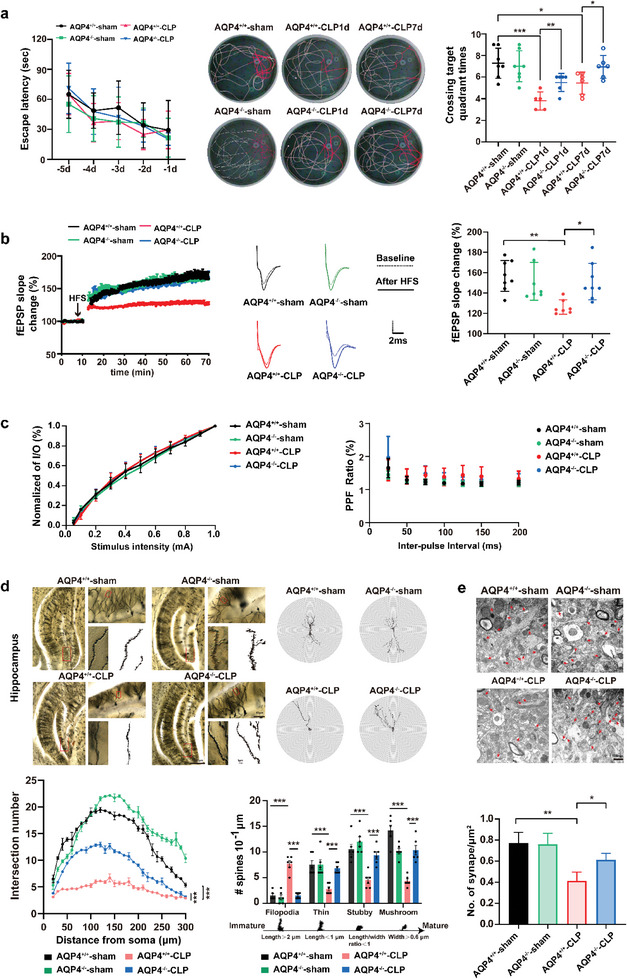
AQP4 knock out ameliorated cognitive dysfunction and improves synaptic plasticity of CLP‐induced sepsis in mice. a) Mice were subjected to the Morris water maze test. Left, the mean escape latency; middle, tracings of the typical swim patterns; right, crossing target quadrant times by the mice. *n* = 5–7 mice for each group. b) Left, the effects of HFS on the fEPSP initial slope (HFS, high frequency stimulation. *n* = 7–8 mice per group). Middle, representative fEPSP traces for data shown. Right, cumulative data showing the mean fEPSP slope 60 min post‐HFS. *n* = 7–8 mice per group. c) Left, cumulative data showing the normalized I/O. Right, cumulative data showing the PPF ratio. *n* = 5 mice per group, 4–5 slices per animal. d) Upper panel, representative dendritic spines in hippocampus of four groups (scale bar, 500 µm, 50 µm, 1 µm); lower left panel, AQP4 knockout in septic mice increases apical node and spines in hippocampus, while AQP4^+/+^‐CLP shows no such change (at least 10 neurons from six mice per group were analyzed by the Sholl); lower right panel, statistical analysis showed the effect of AQP4 knockout in septic mice on dendritic spines. e) Upper panel, representative transmission electron micrographs of synapses in cortex (red arrows: synapses; scale bar, 500 nm); lower panel, statistical analysis of the densities of synapses. Data are presented as mean ± SD. Data in a (Left) was analyzed by repeated‐measures ANOVA with Tukey's post hoc test. a) (Right) One‐way ANOVA with Tukey's post hoc test. b–e) Two‐way ANOVA with Tukey's post hoc test. * *p <* 0.05, ** *p <* 0.01, *** *p <* 0.001, **** *p <* 0.0001.

**Figure 10:**
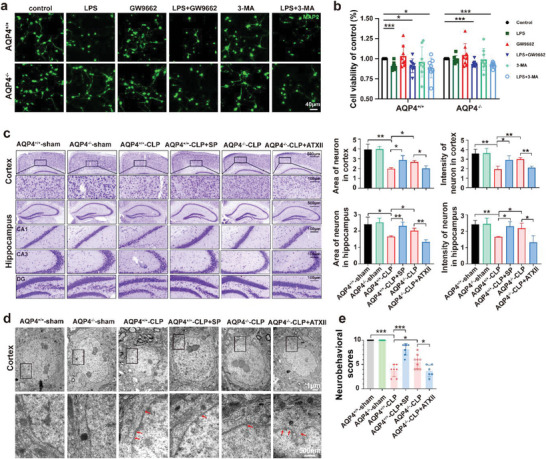
AQP4 knockout antagonized PPAR‐*𝛾* to alleviate neuronal injury via Nav1.6 activation. a) Primary neurons were immunostained with MAP2 (green). The neurons were stimulated with cell culture media of AQP4^+/+^ and AQP4^−/−^ primary astrocytes were treated with GW9662, 3‐MA followed by LPS challenge or not. scale bar, 40 µm. b) CCK8 assay was used to detect the neuron viability of each group. Survival rate = (mean absorbance of experimental group/mean absorbance of control group) × 100%. *n* = 8. c) Representative images of Nissl‐stained sections of cortex and hippocampus from different groups. Scale bar, 500 µm, 100 µm. Right panel, quantification of area and intensity of neuron in the mice cortex and hippocampus among different groups. *n* = 3 mice for each group. d) Cortical neurons of the six treatment groups, visualized by TEM. TEM analysis showed nuclear pyknosis and nuclear membrane rupture (red arrows) in AQP4^+/+^‐CLP, AQP4^+/+^‐CLP+SP, AQP4^−/−^‐CLP, AQP4^−/−^‐CLP+ATX II. Scale bar, 1 µm, 500 nm. e) The neurobehavioral scores of different group mice. *n* = 6–9 mice for each group. Data are presented as mean ± SD. * *p <* 0.05, ** *p <* 0.01, *** *p <* 0.001, one‐way ANOVA with Tukey's post hoc test.

