# Supplementary material for: AQP4 Aggravates Cognitive Impairment in Sepsis‐Associated Encephalopathy through Inhibiting Nav1.6‐Mediated Astrocyte Autophagy
Source: Adv Sci (Weinh). 2023 Mar 15;10(14):2205862. doi: 10.1002/advs.202205862 (PMC10190498; doi:10.1002/advs.202205862)
Supplement: Supplementary file 1 — Supporting Information [file ADVS-10-2205862-s001.pdf]

Table S1 Participant Characteristics

Characteristics	Sepsis group（n=27）	SAE group（n=33）	P
Sex,（male/female）	10/17	20/13	0.069
Age, median (IQR), y	70（21-90）	76（40-90）	0.144
Temperature, °C	37.76±1.13	37.65±1.21	0.719
Heart rate, /min	105.4±21.27	98.48±19.97	0.202
Shock（yes/no）	11/16	18/15	0.287
APACHEII score[a]	16.74±6.24	21.42±6.46	0.006
SOFA score[b]	7.30±4.39	8.36±4.23	0.343
Length of ventilator, d	5.68±4.60	76.2±182.5	0.049
Length of ICU stay, median (IQR), d	12（5-26）	16（8-38）	0.005
Mortality at 28 d, No. (%)	9（33.3%）	22（66.7%）	0.010
Mortality at 60 d, No. (%)	12（44.4%）	22（66.7%)	0.084

Table S1: Abbreviations: IQR, interquartile range.

[a]The Acute Physiology and Chronic Health Evaluation (APACHE) II score ranges from 0 to 71, with higher scores indicating greater risk of hospital death. A score of 25 indicates a mortality probability of approximately 50%.

[b]The Sequential Organ Failure Assessment (SOFA) score ranges from 0 to 24, with higher scores indicating greater severity of organ dysfunction. SOFA scores between 7 and 9 are associated with a 40% to 50% mortality risk.

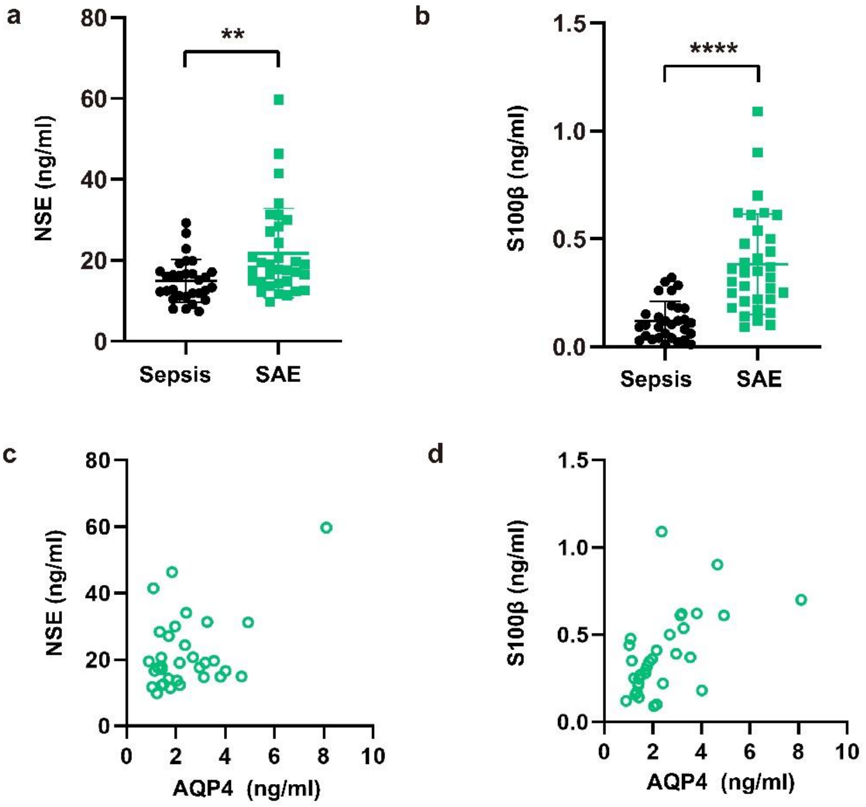

S1. NSE, S100β was elevated in peripheral blood of patients with sepsis associated encephalopathy. a, b. The change of NSE, S-100β levels in peripheral blood of sepsis patients and SAE patients were detected by ELSA. Sepsis patients without encephalopathy (n=27), sepsis related encephalopathy (n=33). Data are represented as the mean ± SD. $^{**}p<0.01$, $^{****}p<0.0001$, Mann–Whitney test. **c, d.** Pearson correlation analysis between AQP4 and NSE, S-100β in patients with sepsis associated encephalopathy.

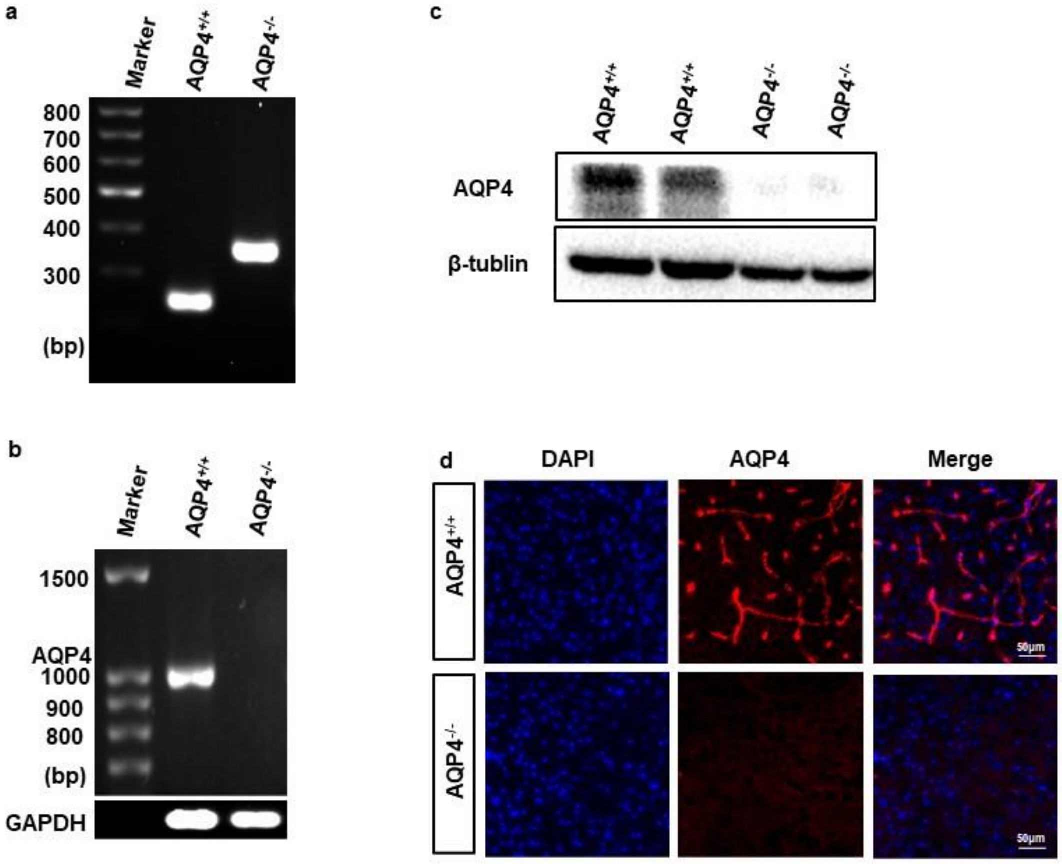

S2. The genotyping of AQP4$^{+/+}$ and AQP4$^{-/-}$ mice at gene level, protein level and morphological level. a. Genotyping of AQP4$^{+/+}$ and AQP4$^{-/-}$ mice. The size of the AQP4$^{+/+}$ amplicon is 240 bp, and the size of the AQP4$^{-/-}$ amplicon is 320 bp. **b.** The mRNA levels of AQP4 were determined by PT-PCR in AQP4$^{+/+}$ and AQP4$^{-/-}$ mice. AQP4$^{-/-}$ mice have no band on the agarose gel. **c.** The representative Western blot band of AQP4 in AQP4$^{+/+}$ and AQP4$^{-/-}$ mice. AQP4$^{-/-}$ mice have no AQP4 protein band. **d.** Representative immunofluorescence images of AQP4$^{+/+}$ and AQP4$^{-/-}$ mice. AQP4$^{+/+}$ and AQP4$^{-/-}$ mice were stained with anti-AQP4 antibody(red), with nuclei stained with DAPI (blue). Scale bar, 50μm.

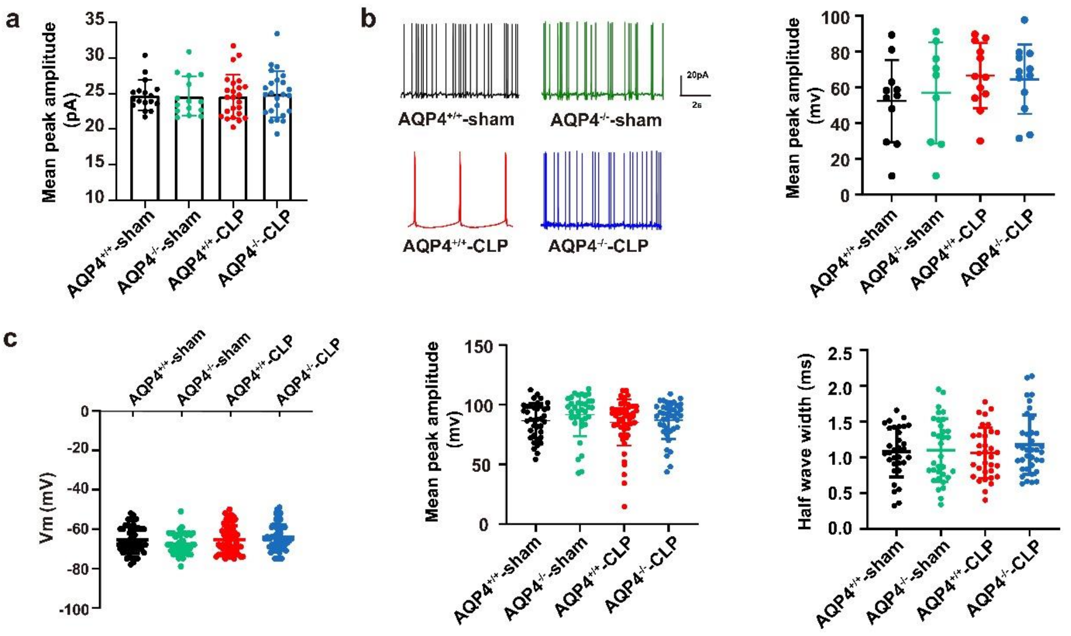

S3. No significant different data of electrophysiology and recording. a. Quantification of the mean peak amplitude of sEPSC. Neurons from 6 mice. **b.** Representative sAP of different groups (*left*) and quantification of sAP mean peak amplitude (*right*). Neurons from 6 mice. **c.** Quantification of eAP membrane potential(*left*) and quantification of the mean eAP peak amplitude(*middle*), quantification of the eAP half wave width (*right*). Neurons from 6 mice for each group. Data are presented as mean ± SD. There were no statistically significant differences among groups. $p>0.05$, two-way ANOVA with Tukey's post hoc test.

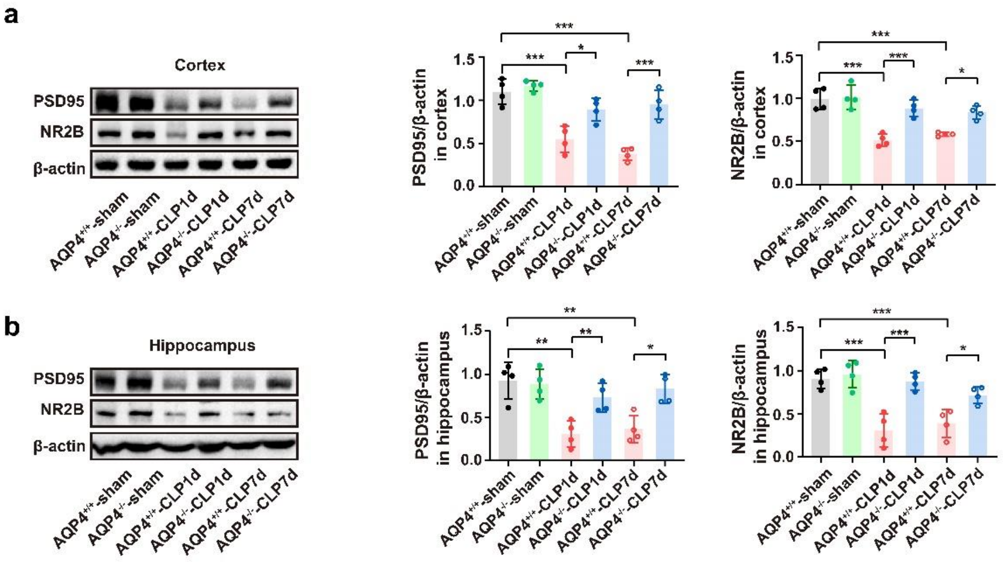

S4. AQP4 knockout alleviate the decline of cognitive function-related molecules in septic mice. a. Expressions of PSD95 and NR2B in cortex were analyzed by western blots. n=4 mice for each group. **b.** Expressions of PSD95 and NR2B in hippocampus were analyzed by western blots. n=4 mice for each group. Data are presented as mean ± SD. * p<0.05, ** p<0.01, *** p<0.001, one-way ANOVA with Tukey's post hoc test.

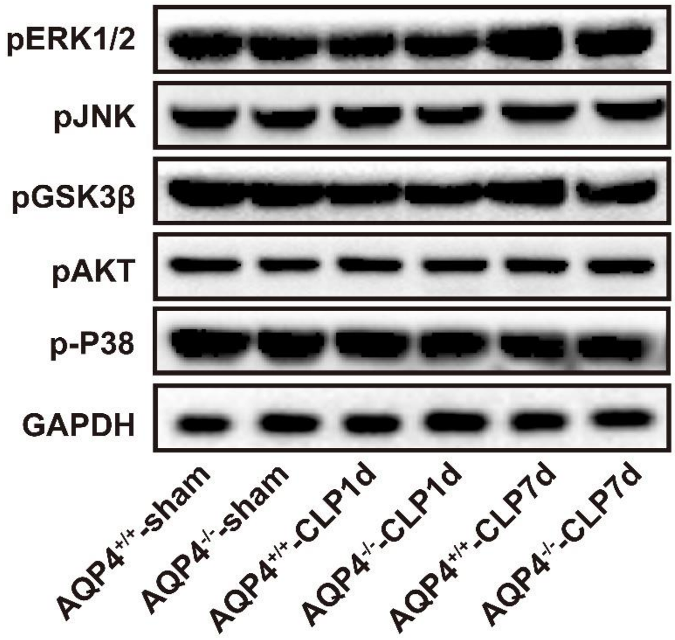

S5. AQP4 has no regulatory effect on other autophagy signaling pathways. Representative Western blot band for p-ERK1/2, pJNK, pGSK3β, pAKT, p-P38.

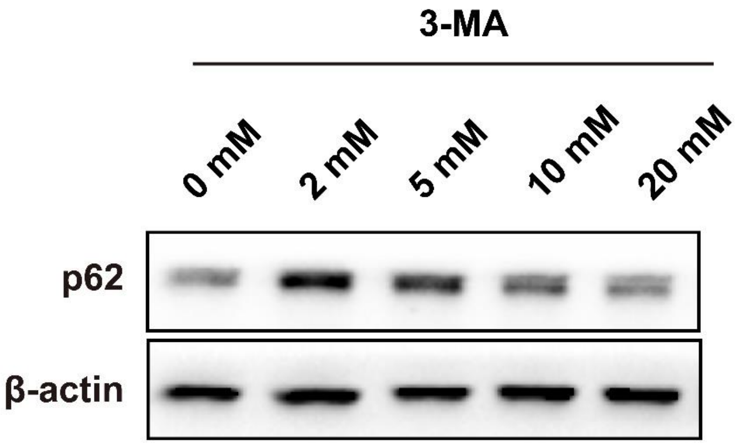

S6. 3-MA drug concentration fumble. Representative bands of p62 protein expression levels of primary astrocytes treated with 3-MA at different concentrations. At 2μM, the expression level of p62 was the highest.

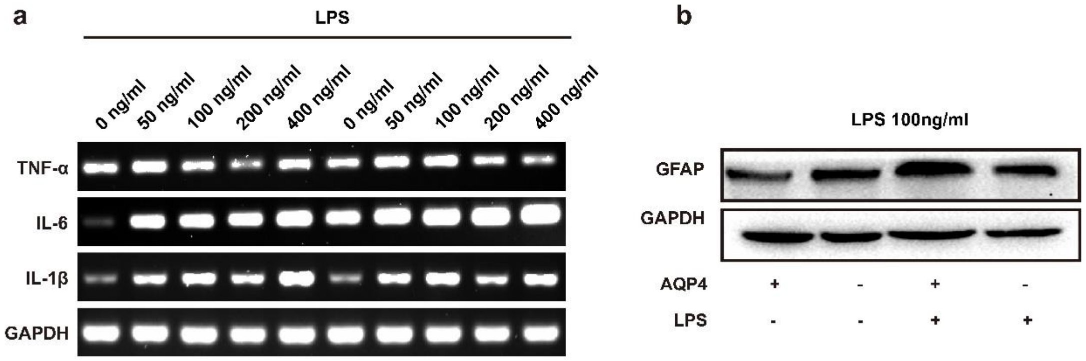

S7. To explore LPS stimulated primary astrocyte concentration. a. Representative bands of TNF-α, IL-6 and IL-1β mRNA expression levels in astrocytes which stimulated by different concentrations of LPS. **b.** Representative Western blot band of GFAP of primary astrocytes treated with 100ng/ml LPS. The GFAP expression level of $AQP4^{+/+}$-LPS significantly increased, while AQP4 knockout, the GFAP expression level was decreased.
